# New insights into the Shwachman-Diamond Syndrome-related haematological disorder: hyper-activation of mTOR and STAT3 in leukocytes

**DOI:** 10.1038/srep33165

**Published:** 2016-09-23

**Authors:** Valentino Bezzerri, Antonio Vella, Elisa Calcaterra, Alessia Finotti, Jessica Gasparello, Roberto Gambari, Baroukh Maurice Assael, Marco Cipolli, Claudio Sorio

**Affiliations:** 1 Department of Medicine, Unit of General Pathology, University of Verona, Italy; 2 Regional Shwachman-Diamond Centre, Cystic Fibrosis Centre, Azienda Ospedaliera Universitaria Integrata di Verona, Italy; 3 Unit of Immunology, Azienda Ospedaliera Universitaria Integrata di Verona, Italy; 4 Department of Life Science and Biotechnology, University of Ferrara, Italy; 5 Department of Pulmonology, Adult CF center, IRCCS Fondazione Cà granda Policlinico Milano, Italy.

## Abstract

Shwachman-Diamond syndrome (SDS) is an inherited disease caused by mutations of a gene encoding for SBDS protein. So far little is known about SBDS exact function. SDS patients present several hematological disorders, including neutropenia and myelodysplastic syndrome (MDS), with increased risk of leukemic evolution. So far, the molecular mechanisms that underlie neutropenia, MDS and AML in SDS patients have been poorly investigated. STAT3 is a key regulator of several cellular processes including survival, differentiation and malignant transformation. Moreover, STAT3 has been reported to regulate neutrophil granulogenesis and to induce several kinds of leukemia and lymphoma. STAT3 activation is known to be regulated by mTOR, which in turn plays an important role in cellular growth and tumorigenesis. Here we show for the first time, to the best of our knowledge, that both EBV-immortalized B cells and primary leukocytes obtained from SDS patients present a constitutive hyper-activation of mTOR and STAT3 pathways. Interestingly, loss of SBDS expression is associated with this process. Importantly, rapamycin, a well-known mTOR inhibitor, is able to reduce STAT3 phosphorylation to basal levels in our experimental model. A novel therapeutic hypothesis targeting mTOR/STAT3 should represent a significant step forward into the SDS clinical practice.

Shwachman-Diamond Syndrome (SDS) is an autosomal recessive disease caused by mutations affecting the Shwachman-Bodian-Diamond syndrome (*SBDS*) gene^1^, which encodes for the SBDS protein, whose exact function is still unknown. SDS is very rare, considering that it affects 1/168,000 newborns in Italy with a mean of 3.0 new cases/year^2^. It has been reported that human SBDS protein is enriched in nucleolus and it seems to be associated with the ribosomal RNA (rRNA) biogenesis^3^. Thus, SDS is considered a ribosomopathy^4^. Consistently with this observation, it has recently postulated that SBDS together with elongation factor-like 1 (EFL1) are involved in the removal of eukaryotic initiation factor 6 (eIF6) during the maturation of the pre-60S ribosomal subunit, allowing the formation of the 80S ribosome^5^. The pathology is characterized by a multiple-organ impairment involving bone marrow dysfunctions, exocrine pancreatic insufficiency, skeletal malformations, hepatic and cognitive disorders^6^. SDS patients present severe hematologic disorders. Neutropenia and impaired neutrophil chemotaxis contribute to recurrent infections in young children^7^. Notably, SDS patients have also an increased propensity for bone marrow failure (about 15% of the cases) and leukemia, in particular acute myeloid leukemia (AML) described in 11% of the patients present in the French Severe Chronic Neutropenia Registry^8^. The progression through AML has been hypothesized as a pro-leukemic effect of SBDS mutations which promotes karyotype instability that in turn leads to clonal anomalies in bone marrow cells^9^. Nevertheless, the same authors suggested that the evolution through AML could be secondary to the acquisition of other mutations in the bone marrow cells^10^. However, the exact pathogenic mechanism whereby defects in ribosome biogenesis could lead to SDS-related neutropenia and myelodysplasia/AML remains unclear.

The mammalian target of rapamycin (mTOR) is a serine/threonine kinase that belongs to the phosphoinositide 3 (PI3K)-related kinase family and resides in at least two multi-protein complexes, namely m-TORC1 and m-TORC2^11^. The m-TORC1 promotes rDNA transcription increasing the activity of RNA polymerase I and leads to rRNA processing to its mature form^12^. The m-TORC1 complex is known to be activated by several MAP kinases such as AKT and ERK1/2, which are able to inhibit the m-TOR endogenous suppressor tuberin (TSC2)^13^. Furthermore, it has been reported that Ras/ERK signalling leads to the phosphorylation of the mTOR-associated Raptor scaffolding protein, which in turn positively regulate mTORC1^14^. Interestingly, 50–80% of AML patients show a constitutive activation of the pathway PI3K/mTOR^15^. However, the role of mTOR in ribosome biogenesis disorders, in particular within SDS pathology, remains poorly elucidated. The signal transduction of mTOR has been also associated to the JAK-STAT signalling. Signal Transducer and Activator of Transcription (STAT) proteins and their activating Janus Kinases (JAK) were originally identified as pathways that mediate interferon signalling^16^. Currently, further insights into the biological roles of JAK-STAT pathways have been raised, stressing STATs as essential regulators of cell proliferation, differentiation and survival in different cellular and animal models^17^. The critical role of JAK-STAT signaling has also been proven in malignant transformation and oncogenesis^18^. The STAT family of transcription factors includes different isoforms: STAT1, STAT2, STAT3, STAT4, STAT5A, STAT5B and STAT6. Reduced mTOR gene expression by small interfering RNA resulted in suppression of STAT3 phosphorylation and decreased production of IFN-γ after IL-12 stimulation in human T cells^19^. The incubation of T cells with rapamycin, a well-known mTOR inhibitor, resulted in a decreased recruitment of STAT3 and phospho-c-Jun to IFN-γ promoter region inhibiting gene transcription^19^. Interestingly, STAT3 dysregulation is also already known to induce AML, playing a role in promoting cell proliferation and survival^20^,^21^. IL-6 is the most common cytokine able to activate STAT3 through JAK1 and JAK2 triggering, leading different blood malignancies^21^. Notably, it has been shown that LPS-stimulated mononuclear cells derived from bone marrow of SDS patients released an increased level of IL-6 into the supernatant culture compared to control subject derived cultures^22^. Furthermore, IL-6-dependent STAT3 activation may induce expression of inflammatory cytokines, among which IL-6 itself, generating a loop which further induces the JAK/STAT3 pathway^23^. Besides its role in the malignant transformation, STAT3 has been identified as a critical regulator of neutrophil development and granulopoiesis during granulocyte-colony stimulating factor (G-CSF) stimulation^24^,^25^. Here we show for the first time, to the best of our knowledge, that SDS patients present a dysregulation of both mTOR and STAT3 pathways, due to a constitutive activation of MAPK ERK1/2. Currently, several drugs approved by US Food and Drug Administration (FDA) and European Medicine Agency (EMA) targeting the JAK–STAT pathway are available for the treatment of different forms of leukemia and lymphoma. In this respect, the JAK1-JAK2 inhibitor Ruxolitinib has been already approved by FDA for the treatment of myeloproliferative diseases^21^. Several m-TORC and dual PI3k-mTOR inhibitors have been developed and are currently being evaluated within clinical trials for the treatment of several malignancies, including AML^26^. Our findings suggest that mTOR inhibitors could be helpful in SDS pathology as well, opening a wider scenario within the current therapeutic approaches. The aim of the present study was to gain further insights into the molecular mechanisms that underlie the haematological disorders observed in patients affected by SDS, in order to find novel molecular targets, which might be helpful to develop new therapeutic strategies.

## Results

### Expression of SBDS protein in Lymphoblastoid cell lines (LCLs)

Currently, SDS cell models are still poorly developed, although pluripotent stem cell models of SDS through knockdown of SBDS in human embryonic stem cells and induced pluripotent stem cell (iPSC) lines from two SDS patients has been previously reported^27^. Lymphoblasts are immature cells that typically differentiate to form mature lymphocytes. Epstein-Barr virus infection is able to transform mature B cells into lymphoblastoid cell lines (LCLs) that have been reported to proliferate and expand almost indefinitely. Somatic mutation rate in LCLs is very low, about 0.3%^28^ allowing to conclude that LCLs can be used to perform genetic and proteomic analysis. We obtained five different LCL lines derived from SDS patients carrying the most common mutations of SBDS gene (258 + 2T > C and 183-184TA > CT), namely LY-190, LY-193, LY-198, LY-222 and LY-223 and three different cell lines from healthy donors, namely LY-52, LY-53 and LY-M. The expression of SBDS protein has been tested in these lymphoblastoid cells by western blot analysis. The results obtained indicate that the 28.8 KDa SBDS protein was expressed in LY-52 and LY-53 control cell lines and undetectable in LY-190, LY-193 and LY-198 SDS cell lines (see Supplemental Figure S1).

### Analysis of phospho-kinases and STATs activation in LCLs upon IL-6 stimulation

**Figure 1:**
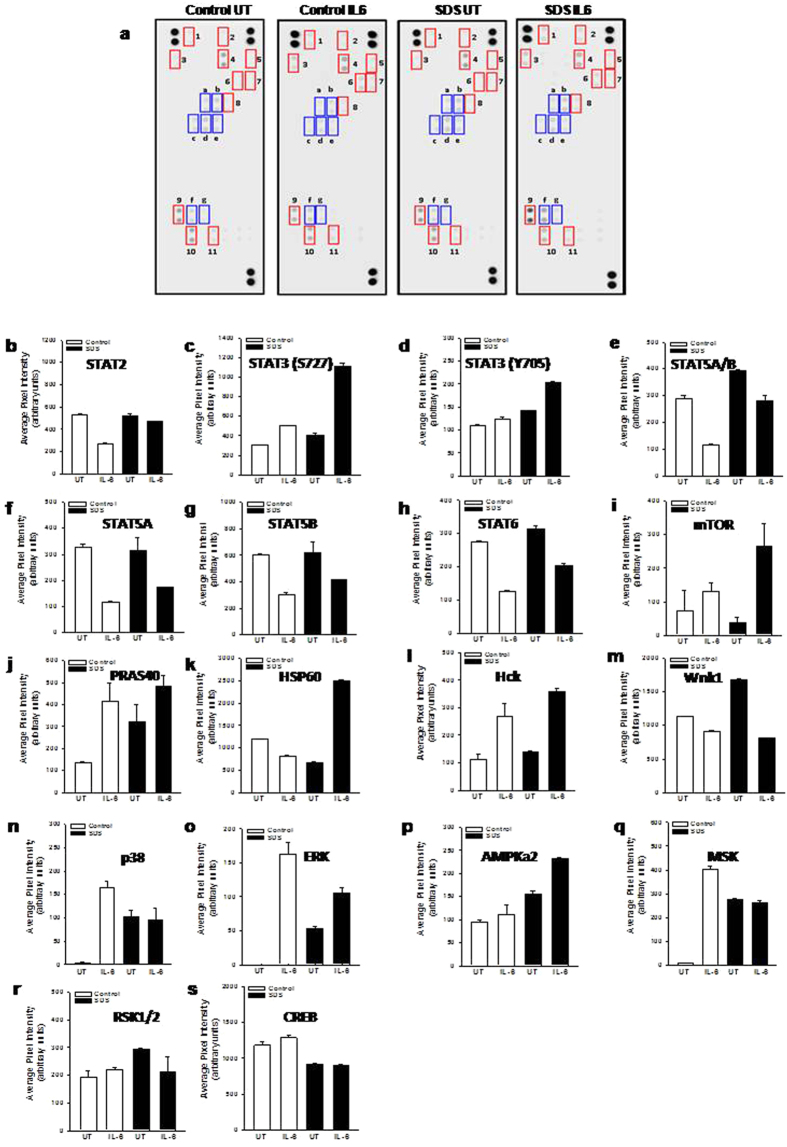
Human Phospho-kinase array. A pool of 450 μg (150 μg each) of LY52, LY53 and LYM cell lysates (Control) or a pool of 450 μg (150 μg each) of LY190, LY193 and LY198 cell lysates (SDS) in the presence or in the absence (UT) of IL-6 (10 ng/ml) were incubated in nitrocellulose membrane pre-spotted with 43 antibodies able to recognize 43 different phospho-kinases. A cocktail of biotinylated detection antibodies was added followed by streptavidin-HRP incubation. Chemiluminescent detection reagents were applied and a signal was produced at each capture spot corresponding to the amount of phosphorylated protein bound. (**a**) Scanning of the arrays in which there are highlighted: i) red boxes, representing the major activated kinases; ii) blue boxes, representing phosphorylated STATs; **1**, Hck; **2**, mTOR; **3**, PRAS40; **4**, CREB; **5**, p38; **6**, MSK; **7**, ERK; **8**, AMPK2a; **9**, HSP60; **10**, Wnk1; **11**, RSK; (**a)**, STAT6; (**b**) STAT2; (**c**) STAT5A/B; (**d**) STAT5B; (**e**) STAT5A; (**f**) STAT3 (S727); (**g**) STAT3 (Y705). (**b–s)** quantitative analysis of pixel densities on developed X-ray film of the major regulated proteins.

IL-6 is a pro-inflammatory cytokine able to promote STAT3 activation through JAK1 and JAK2 phosphorylation^29^. Moreover, IL-6 has been reported to act as a differentiation factor on hematopoietic cells and a pro-proliferative stimulus for B cells acting via STAT3 activation^30^. In order to verify the possible presence of a comprehensive mechanistic model of IL-6-dependent STAT3 hyper-activation in SDS LCLs, we tested the phosphorylation level of 43 different kinases and STATs proteins by a Human Phospho-Kinase array, as previously described^31^. Healthy control- and SDS-derived LCLs were pre-incubated for 30 min in the presence or in the absence of human recombinant IL-6 (10 ng/ml) and subjected to western-blot like analysis using nitrocellulose membrane pre-spotted with specific antibodies (Fig. 1a). Interestingly, STAT3 was the only STAT isoform showing a significant activation upon exposure to IL-6. In fact, increased phosphorylation levels of both serine S727 and tyrosine Y705 residues have been detected (Fig. 1c,d). Notably, we found that IL-6-dependent STAT3 phosphorylation level of both S727 and Y705 is higher in SDS LCLs than in LCLs expressing wild-type SBDS (Fig. 1c,d). In particular, phosphorylation of S727 seems to be greatly enhanced in SDS cells. On the contrary, we found a reduced IL-6-dependent STAT5A/B and STAT6 phosphorylation (Fig. 1e–h). Among the 43 different kinases tested, we found IL-6-dependent increase in phosphorylation of mTOR, PRAS40, Hck, ERK1/2 and AMPKα2 both in SDS LCLs and control LCLs (Fig. 1i–p), but SDS cells generally reported higher responsiveness to IL-6 than healthy control cells. HSP60 phosphorylation was induced by IL-6 only in SDS LCLs (Fig. 1k). Furthermore, p38 (Fig. 1n), ERK1/2 (Fig. 1o), AMPKα2 (Fig. 1p) and MSK1/2 kinases (Fig. 1q) showed a constitutive hyper-activation in SDS LCLs. We reported also a reduction of phosphorylation of Hck (Fig. 1l) and Wnk1 (Fig. 1m) upon stimulation with IL-6 both in SDS- and healthy subject-derived LCLs. Finally, we found no appreciable changes in phosphorylation levels of RSK kinase and its downstream regulated transcription factor CREB upon exposure to IL-6 (Fig. 1r,s).

### LCLs obtained from SDS patients show hyper-activation of mTOR and STAT3

**Figure 2:**
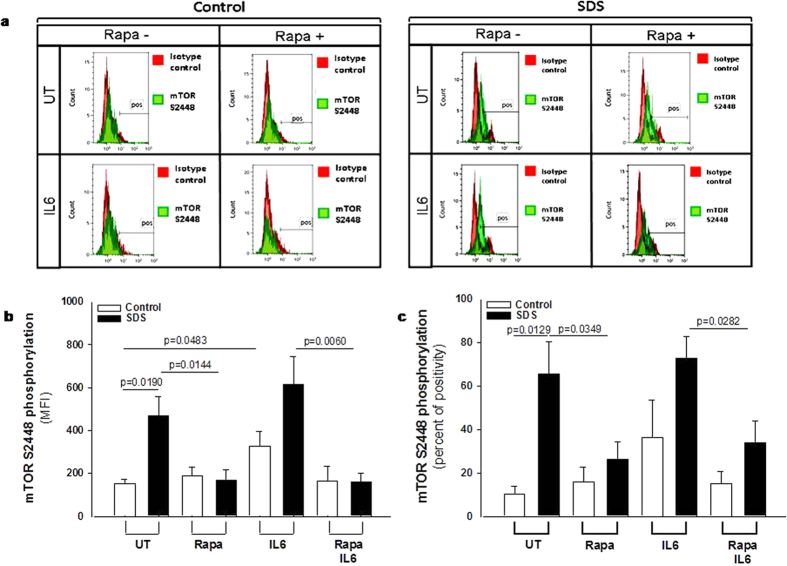
Flow cytometric analysis of mTOR S2448 phosphorylation in LCLs. **(a**) Representative experiment indicating mTOR S2448 phosphorylation level (green histogram) in healthy donor derived LCLs (Control) versus SDS LCLs (SDS). Red histogram indicates isotype control. Control LCLs and SDS LCLs were pre-incubated with 350 nM rapamycin (Rapa) for 1 hour before stimulation in the presence or in the absence (UT) of IL-6 (10 ng/ml) for further 15 min. (**b**) Median Fluorescence Intensity (MFI) and Percent of positive cells **(c)** derived from five independent experiments performed in LCLs derived from five different SDS patients. Data are mean ± SEM. Student’s t-test has been calculated.

**Figure 3:**
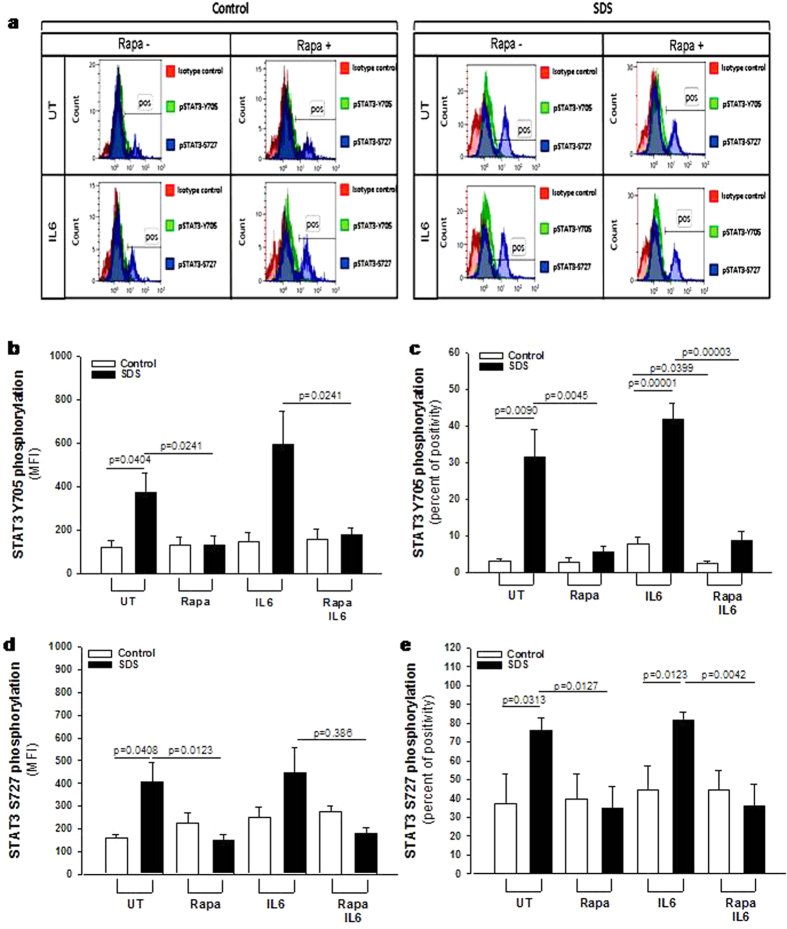
Flow cytometric analysis of STAT3 Y705 and S727 phosphorylation in LCLs. Representative experiment indicating STAT3 Y705 (green histogram) and S727 (blue histogram) phosphorylation level in: (**a**) healthy donor derived LCLs (Control) versus SDS LCLs (SDS). Red histogram indicates isotype control. Control LCLs and SDS LCLs were pre-incubated with 350 nM rapamycin (Rapa) for 1 hour before stimulation in the presence or in the absence (UT) of IL-6 (10 ng/ml) for further 15 min. (**b**) Median Fluorescence Intensity (MFI) and Percent of positive cells **(c)** for STAT3 Y705 signal. (**d**) Median Fluorescence Intensity (MFI) and Percent of positive cells **(e)** for STAT3 S727 signal. Data are mean ± SEM of five independent experiments performed in LCLs derived from five different SDS patients. Student’s t-test has been calculated.

**Figure 4:**
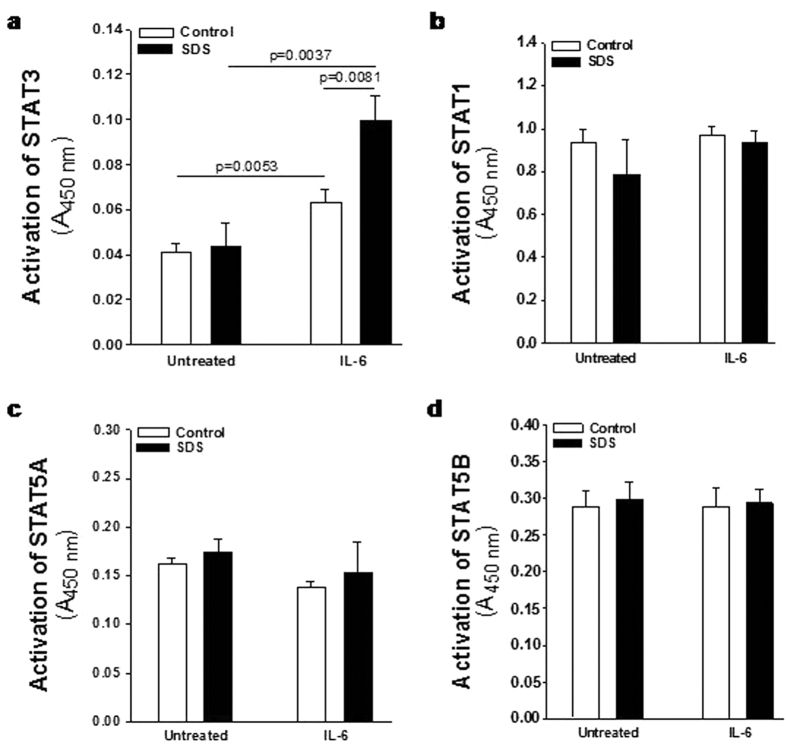
IL-6-dependent nuclear translocation of STAT3 in LCLs. Cells have been challenged with IL-6 (10 ng/ml) for 15 min. Human Phospho STAT Family Trans-AM kit was performed using 2.5 μg nuclear extracts for each sample. Histograms represent the nuclear translocation of: (**a**) STAT3; (**b**) STAT1; (**c**) STAT5A; (**d**) STAT5B. Data are mean ± SEM of 4 experiments performed in 3 different SDS cell lines versus 3 different healthy control cell lines, in duplicate. Mann-Whitney test has been reported.

In order to verify the data obtained from the phospho-kinase arrays, we performed a phospho flow analysis^32^ of IL-6-dependent mTOR and STAT3 activation in LCLs. mTOR exists in two different complexes, mTOR complex 1 (mTORC1), which is rapamycin sensitive, and mTORC2, which become rapamycin sensitive only after prolonged exposure to the inhibitor. mTOR is mainly phosphorylated at S2448 in mTORC1^33^ through S6K1 kinase pathway^34^. We compared IL-6-dependent phosphorylation of mTOR on its S2448 site, by phospho flow and expressed as Median Fluorescence Intensity (MFI) (Fig. 2b) and % of positive cells (Fig. 2c) in EBV-transformed B cells derived from three different SDS patients and healthy controls. Results indicate a constitutive pre-activation in SDS samples, whereas IL-6 treatment further enhanced the signal. The increase of S2448 phosphorylated mTOR in SDS samples was also confirmed by western-blot analysis (Supplementary Fig. S2a,b). Furthermore, pre-incubation with the specific mTOR inhibitor rapamycin (Sirolimus) is capable to restore normal level of mTOR activation in SDS-derived LCLs (Fig. 2b,c). The rapamycin-mediated decrease of S2448 phosphorylated mTOR in control LCLs and SDS-derived LCLs was also demonstrated by western-blot analyses using samples stimulated or not with IL-6 (Supplementary Fig. S2c,d). STAT3 pathway was also dysregulated in LCLs obtained from SDS patients (Fig. 3). Both Y705 and S727 phosphorylation sites of STAT3 have been assessed by phospho flow analysis in five different LCLs obtained from SDS patients. Results indicated that SDS transformed B cells present a 2-fold increased constitutive phosphorylation of STAT3 in Y705 (Fig. 3b,c) and S727 (Fig. 3d,e) expressed as MFI and percentage of positive cells. Since it has been previously reported that inhibition of mTOR by rapamycin reduces STAT3 phosphorylation in IL-12-treated human T cells^19^, we tested the effect of rapamycin in our experimental model. The results obtained indicate that low concentration (350 nM) of rapamycin is able to strongly inhibit the constitutive STAT3 hyper-phosphorylation observed both in Y705 and S727 residues (Fig. 3). STAT3 is a transcription factor which, once activated through phosphorylation in Y705 and S727, dimerize and translocate into the nucleus to control target gene expression. Importantly, STAT3 activation and nuclear translocation plays a key role both in neutrophil development and in AML transformation^35^. In order to verify whether STAT3 hyper-phosphorylation correspond to an increased nuclear translocation, we performed a specific Trans-AM assay able to detect nuclear localization of STAT1, STAT3, STAT5A and STAT5B isoforms on nuclear extracts of LCLs in the presence or in the absence of IL-6 stimulation. Both SDS and healthy control cells showed a significant increase in nuclear translocation of STAT3 upon IL-6 stimulation, which was significantly higher in SDS cells (Fig. 4a). No detectable difference in nuclear localization was observed in SDS in comparison to healthy control cells in resting condition. None of the other STATs isoforms tested showed increased nuclear localization upon IL-6 stimulation (Fig. 4b–d) suggesting a specific role of STAT3 in SDS.

### Lack of SBDS expression leads to IL-6-dependent mTOR hyper-phosphorylation through ERK1/2 activation

**Figure 5:**
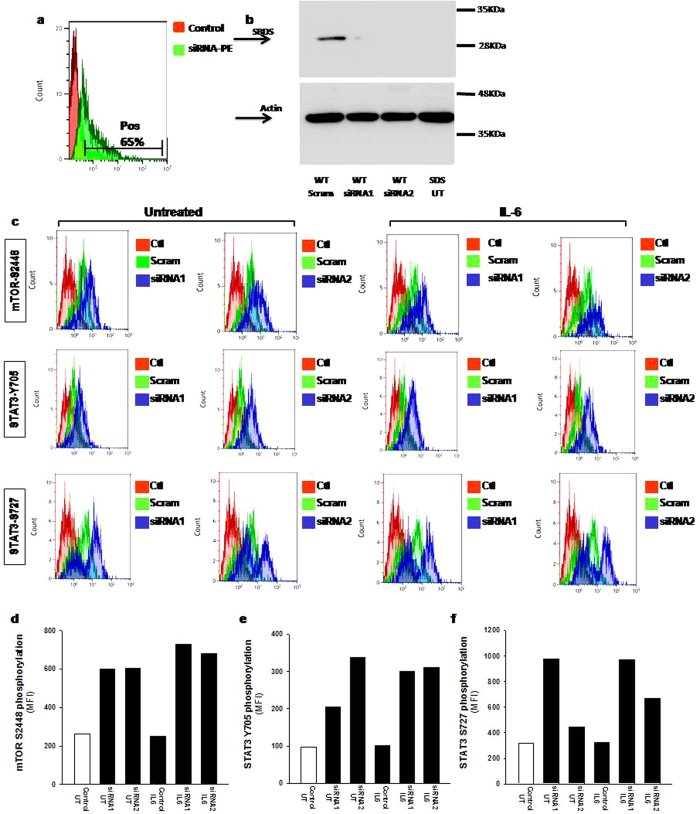
Effect of SBDS gene silencing in healthy donor derived cells on mTOR S2448 phosphorylation. LCLs derived from healthy donors were transiently transfected with 2 different specific siRNA sequences (siRNA 1 and siRNA 2) for SBDS, or with PE-conjugated siRNA sequence, or with scrambled sequence as control in the presence of cationic liposomal vector for 24 hours and stimulated with IL-6 (10 ng/ml) for further 15 min. (**a**) Check of efficiency rate of transfection measured by flow cytometry using a PE-conjugated siRNA. Results indicate up to 65% of transfection efficiency in our cell model. (**b**) Effect of SBDS gene silencing on SBDS protein expression in Control LCLs as measured by western blot analysis (SDS UT are SDS LCLs untreated as internal control). (**c**) Effect of SBDS gene silencing on mTOR S2448, STAT3 Y705 and STAT3 S727 phosphorylation measured byFC. (**d–f**) Median Fluorescence Intensity of phospho-mTOR and phosphor-STAT3 signals.

In order to verify whether mTOR hyper-phosphorylation is associated with the loss of SBDS in the absence of other potential concurrent alterations present in SDS cells, we transiently silenced gene expression of SBDS in LCLs derived from healthy donors. To this aim, we transfected specific short interfering (si)RNA molecules in LCLs using a liposomal vector. Since transfection efficiency depends on cell type and experimental conditions, we measured uptake efficiency of our PE-conjugated siRNA into LCLs by flow cytometry (FC). We reported an efficiency transfection rate of 65% (Fig. 5a). To check the down-regulation of SBDS protein expression, we performed western blot analysis. Gene silencing produced a strong decrease of SBDS protein expression in lymphoblastoid cells, reducing SBDS expression at a level very similar to that observed in SDS cells (Fig. 5b). Notably, knock-down of SBDS gene in normal LCLs resulted in a considerable up-regulation of mTOR phosphorylation in S2448 residue as well as of STAT3 phosphorylation, both in Y705 and S727 as measured by FC (Fig. 5c–f). In order to verify this result, a Phospho-mTOR (S2448) ELISA test was performed on SBDS-silenced cells. Results indicated a statistically significant up-regulation (2-fold) of phosphorylation of mTOR S2448 (see Supplemental Fig. S3a), similarly to that observed in SDS condition (Fig. 2b). The m-TORC1 complex is known to be activated by MAP kinases such as ERK1/2, which is able to inhibit the m-TOR endogenous suppressor tuberin (TSC2)^13^. Since we found a constitutive ERK1/2 activation in SDS cells (Fig. 1o), we verified whether MAPK ERK1/2 is involved in our experimental model. In order to address this issue, we measured mTOR (S2448) phosphorylation in LCLs pre-incubated in the presence and in the absence of specific ERK1/2 inhibitor U0126, as previously reported^31^. Pre-incubation with U0126 led to increase in constitutive phosphorylation accompanied by a strong inhibitory effect on IL-6-dependent mTOR S2448 phosphorylation in SDS cells (see Supplemental Fig. S3b).

### Assessment of mTOR and STAT3 hyper-activation in primary leucocytes derived from SDS patients

**Figure 6:**
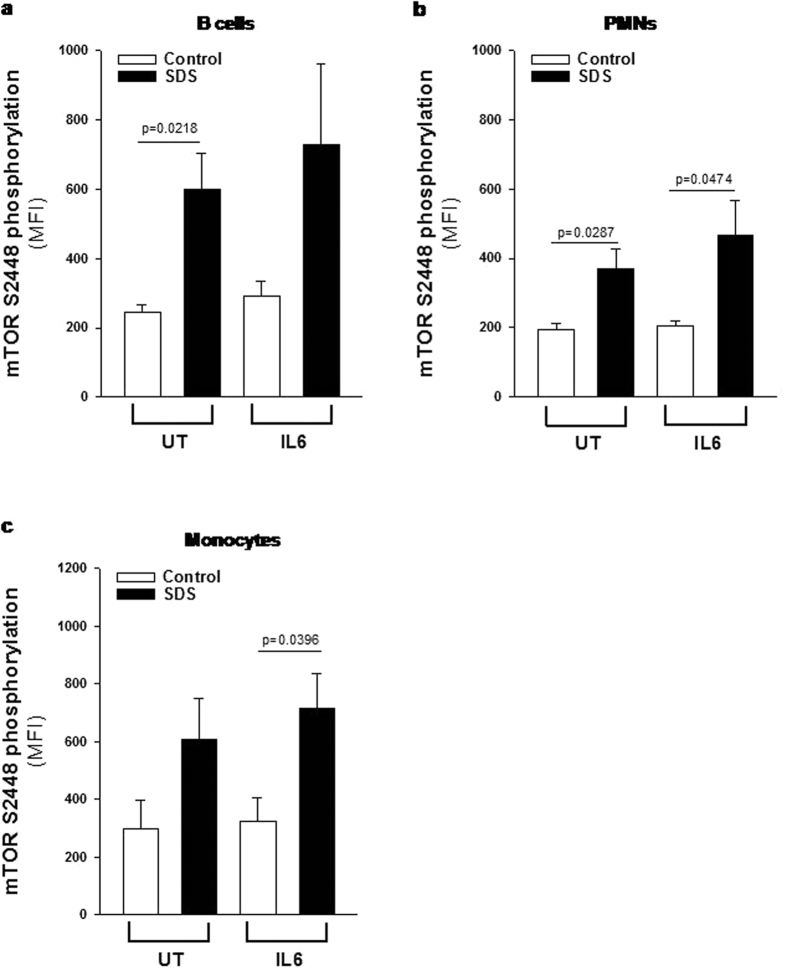
Flow cytometric analysis of mTOR S2448 phosphorylation in primary leukocytes. Primary leukocytes were incubated in the absence (UT) or in the presence of IL-6 stimulation (10 ng/ml) for 15 min and analyzed by flow cytometry. (**a**) MFI of mTOR S2448 phosphorylation measured in primary B cells. (**b**) MFI of mTOR S2448 phosphorylation measured in primary PMNs. (**c**) MFI of mTOR S2448 phosphorylation measured in primary monocytes. Data are mean ± SEM of five independent experiments performed in LCLs obtained from five different SDS patients and compared to five different healthy donors. Student’s t-test has been reported.

**Figure 7:**
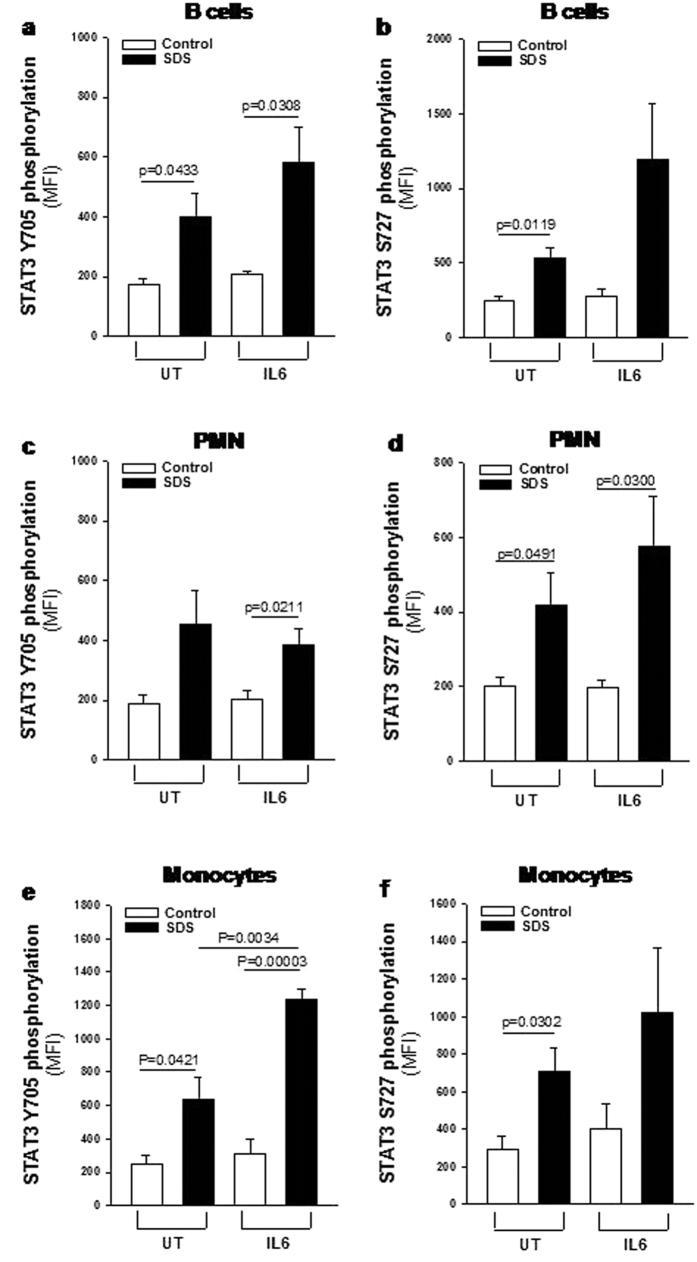
Flow cytometric analysis of STAT3 Y705 and S727 phosphorylation in primary leukocytes. Primary leukocytes were incubated in the absence (UT) or in the presence of IL-6 stimulation (10 ng/ml) for 15 min and analyzed by FC. (**a,b**) MFI of STAT3 Y705 and S727 phosphorylation (respectively) measured in primary B cells. (**c,d**) MFI of STAT3 Y705 and S727 phosphorylation (respectively) measured in primary PMNs. (**e,f**) MFI of STAT3 Y705 and S727 phosphorylation (respectively) measured in primary monocytes. Data are mean ± SEM of five independent experiments performed in LCLs obtained from five different SDS patients and compared to five different healthy donors. Student’s t-test has been reported.

Although LCLs has been reported to maintaining a close similarity to the primary lymphocytes^28^,^36^ we verified the presence of dysregulated phosphorylation of mTOR and STAT3 also in primary leucocytes. In order to address this issue we collected peripheral blood samples from five SDS patients presenting the same genotype of patients from whom we generated LCLs (258 + 2T > C/183-184TA > CT, which are the most common mutations in SDS), and in parallel from healthy donors matched for age and sex. Consistently with results obtained in SDS LCLs, primary B cells, monocytes and PMNs derived from SDS patients showed higher constitutive and IL-6-induced levels of phospho-mTOR (S2448) than those observed in healthy control cells (Fig. 6 and Supplemental Fig. S4). Notably, pre-incubation of primary leukocytes isolated from SDS subjects with 350 nM rapamycin restored mTOR S2448 phosphorylation to normal levels (Supplemental Fig. S5). Furthermore, primary SDS B cells, monocytes and PMNs showed increased phosphorylation of STAT3 both in terms of Y705 and S727 compared to control cells (Fig. 7 and Supplemental Fig. S6), thus fully confirming STAT3 pathway dysregulation in SDS. Again, pre-incubation of primary leukocytes isolated from SDS subjects with 350 nM rapamycin strongly reduced both Y705 and S727 STAT3 phosphorylation (Supplemental Figs S7 and S8), consistently with data derived from LCLs.

### mTOR and STAT3 inhibition differentially affects cell proliferation and apoptosis in LCLs derived from SDS patients compared to healthy donors-derived cells

**Figure 8:**
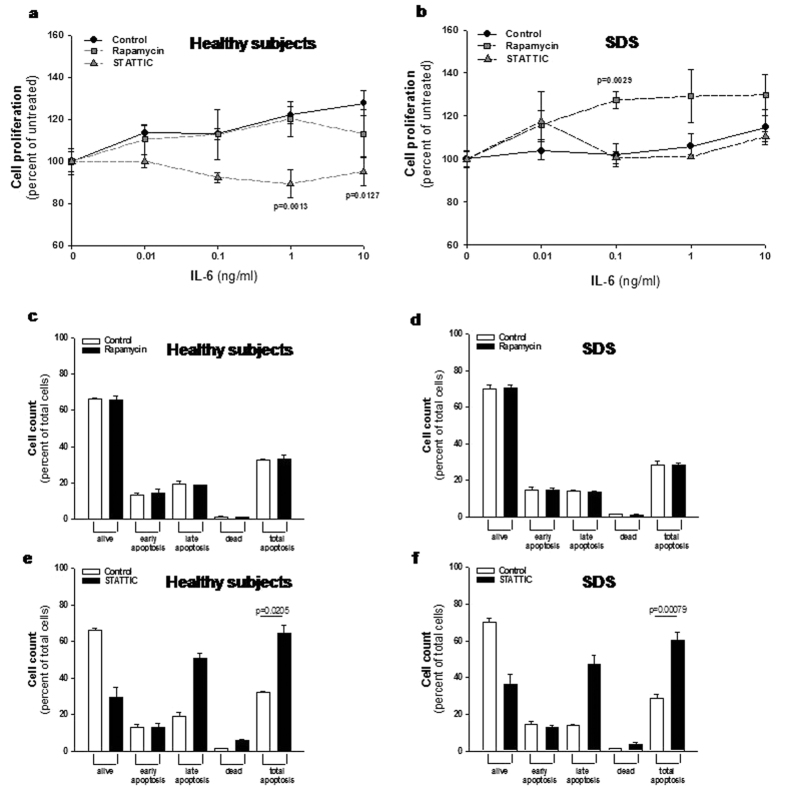
Effect of rapamycin and STATTIC on cell proliferation and apoptosis in LCLs. (**a,b**) LCLs derived from both healthy donors (**a**) and SDS patients (**b**) were incubated in the presence or in the absence of 350 nM mTOR inhibitor rapamycin, or 20 μM STAT3 inhibitor STATTIC and stimulated with increasing doses (0.01–10 ng/ml) of IL-6 for 48 hours. Cell proliferation was measured by XTT Cell Proliferation Kit II. Data are mean ± SEM of five independent experiments performed in duplicate. Student’s t-test has been calculated. (**c,d**) LCLs derived from both healthy donors (**c**) and SDS patients (**d**) were incubated in the presence (black bars) or in the absence (white bars) of 350 nM mTOR inhibitor rapamycin for 24 hours. Apoptosis was analyzed using the Muse Annexin V & Dead Cell Kit. Data are mean ± SEM of four independent experiments performed in duplicate. (**e,f**) LCLs derived from both healthy donors (**e**) and SDS patients (**f**) were incubated in the presence (black bars) or in the absence (white bars) of 20 μM STAT3 inhibitor STATTIC for 24 hours. Apoptosis was analyzed using the Muse Annexin V & Dead Cell Kit. Data are mean ± SEM of four independent experiments performed in duplicate. Student’s t-test has been calculated.

Usually, the effect of rapamycin in most normal cells and tumor cell lines is growth retardation, even though in some tumor cell lines and in primary cells, treatment with rapamycin leads to apoptosis^37^. In order to check the effect of low doses (350 nM) of rapamycin on cell proliferation, we tested LCLs proliferation in the presence or in the absence of this mTOR inhibitor. Besides its role in activating STAT3, IL-6 has been reported to act as a differentiation factor on hematopoietic cells and to induce B-cell proliferation^38^. Thus, we induced LCLs proliferation incubating cells with increasing doses of IL-6 in the presence or in the absence of rapamycin. Results indicated that LCLs derived from SDS patients present a lower IL6-dependent growth rate than healthy control cells (Fig. 8a,b). Interestingly, rapamycin restored SDS cell growth rate to the normal level in our experimental model (Fig. 8b). Since rapamycin has been reported to induce apoptosis in some cell models, we checked this process in LCLs. Incubation of 350 nM rapamycin in LCLs showed no apoptotic effect on both normal and SDS LCLs (Fig. 8c,d). Subsequently, we tested also the effect of STAT3 inhibition on cell proliferation and apoptosis pre-incubating LCLs with 20 μM STAT Three Inhibitory Compound (STATTIC), which has been recently reported to induce apoptosis and block cell proliferation in prostate and colon cancer cells^39^,^40^. The results obtained indicated that STATTIC blocked cell proliferation of normal LCLs, whereas we found no effect on cell proliferation in SDS-derived cells (Fig. 8a,b). As previously reported^39^, apoptosis was strongly induced upon STATTIC treatment both in healthy controls and SDS cells (Fig. 8e,f).

## Discussion

**Figure 9:**
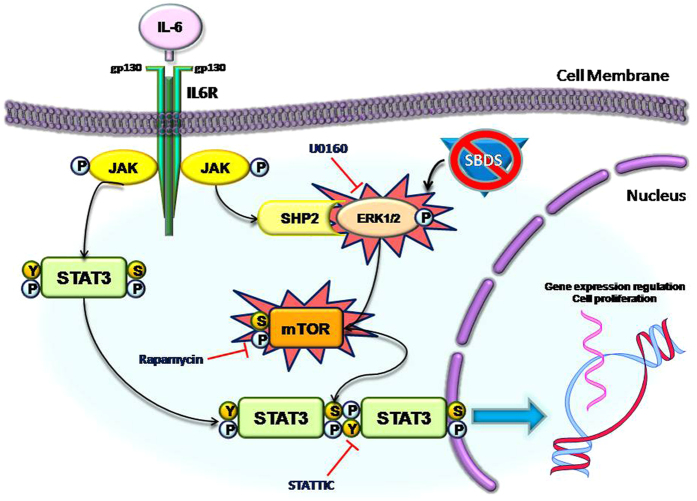
Model of dysregulated mTOR/STAT3 signal transduction pathways observed in leukocytes obtained from SDS patients. Normally, IL-6 trigger a JAK1/2 activation which in turn leads to STAT3 phosphorylation, mainly at Y705 residue, causing STAT3 dimerization and translocation into the nucleus, where STAT3 is able to regulate gene expression orchestrating several cellular processes like inflammation and cell proliferation. In SDS patients, leukocytes show ERK1/2, mTOR and STAT3 hyper-activation. ERK1/2 is known to promote mTOR phosphorylation in S2448 residue, which in turn leads to mTORC1 complex activation. mTORC1 is known to regulate different cell processes, including translation, autophagy, cell growth and ribosome biogenesis, which are impaired in SDS pathology. Notably, mTORC1 is also known to induce strong phosphorylation of STAT3 both in Y705 and S727 residues in different cellular models. Here we report that mTOR inhibitor rapamycin is able to reduce STAT3 hyper-activation observed in SDS patients, restoring phosphorylation level of both Y705 and S727. Moreover, in this issue we show how loss of SBDS protein can lead to mTOR S2448 hyper-activation in LCLs obtained from healthy donors. Finally, we show that pre-incubating ERK1/2 inhibitor U0126 in SDS EBV-transformed B cells we significantly reduce IL-6 induced mTOR S2448 phosphorylation.

Haematological issues including severe neutropenia and myeloid dysplasia, which in turn increases the risk of acute myeloid leukemia development, are the major causes of morbidity and mortality in SDS. However, the molecular mechanisms that underlie these processes remain so far unclear. STAT3 has been reported to play a key role in neutrophil development and granulopoiesis^24^ as well as in AML progression by promoting cell proliferation and survival^20^,^21^. It has been previously reported that mTORC1 complex can phosphorylate STAT3 in both residues Y705 and S727 in several cell models^19^,^41^,^42^,^43^,^44^, and that rapamycin reduces STAT3 transcriptional activity^45^. Interestingly, also mTOR activation may acts as driving force to promote AML transformation, since 50–80% of patients affected by AML present constitutive activation of the PI3K/mTOR pathway^15^. Here we demonstrate that both mTOR and STAT3 pathways are constitutively up-regulated in SDS in LCLs models and confirmed these findings in primary leukocytes. In respect to this issue, we found that mTOR S2448 site is highly phosphorylated and more responsive to IL-6 stimulation in SDS cells than in healthy donor cells. In order to verify whether the loss of SBDS could be associated to this process, we performed gene silencing experiments in normal LCLs, knocking-down SBDS gene using siRNA molecules. Data confirm that loss of SBDS protein in healthy donor derived LCLs reproduces the condition observed in SDS cells by doubling the basal mTOR activation levels, suggesting a key role for SBDS in controlling this pathway. However, at the present time we cannot assert that the loss of SBDS function is directly linked to mTOR/STAT3 hyper-activation and this point will be matter of further investigation. Since S2448 site is the major activator site of mTORC1 complex, which in turn regulates translation, autophagy, cell growth and ribosome biogenesis, this finding may represent a key step towards the understanding of the molecular mechanisms that lead to haematological disorders in SDS. Furthermore, we describe a constitutive activation of MAPK ERK1/2 signalling in SDS LCLs. Since ERK1/2 promotes mTORC1 activation by inhibiting TSC2, which is an endogenous inhibitor of mTOR^13^, we tested the effect of ERK1/2 chemical inhibitor U0126 in SDS LCLs in the presence and in the absence of IL-6 stimulation, resulting in significant decreasing of IL-6 dependent mTOR activation. The results suggest that ERK1/2 basal hyper-activation acts as the driving force to mTORC1 hyper-activation, which in turn keeps turning on the STAT3 signalling pathway by phosphorylating both its Y705 and S727 residues leading to dysregulated gene expression and modifying cell proliferation (summarized in Fig. 9). These findings could contribute to explain the haematological defect observed in SDS patients, providing a molecular basis to understand the elevated risk of developing AML. The relevance of our findings is amplified by the fact that several drugs approved by US Food and Drug Administration (FDA) and European Medicine Agency (EMA) targeting the JAK–STAT pathway are already available for the treatment of different forms of leukemia and lymphoma. The JAK1-JAK2 inhibitor Ruxolitinib has been already approved by FDA for the treatment of myeloproliferative diseases^21^. However, here we show that STAT3 inhibition leads to induction of apoptosis in both normal and SDS cells. The observation that no effect on SDS LCLs proliferation was present upon STATTIC inhibitor treatment might be explained by the fact that these cells have a reduced growth rate compared to normal cells. These results suggest a potential warning on the use of STAT3 inhibitors on SDS patients, who already present increased apoptosis rate in haematological progenitors leading to bone marrow failure^8^. Interestingly, rapamycin showed no effect on LCLs apoptotis instead, but restored SDS IL-6-depenent cell proliferation to normal level, suggesting Sirolimus as a promising molecule for SDS therapy. Furthermore, m-TORC and dual PI3k-m-TOR inhibitors have been developed and are currently being evaluated within clinical trials for the treatment of several malignancies, including AML^26^. Rapamycin analog Everolimus (RAD001) has been already approved by FDA and EMA for the treatment of advanced renal cell carcinoma^46^. Interestingly, RAD001 has been recently tested for the therapy in a Phase I clinical trial on refractory multiple myeloma^47^. A new generation of dual m-TOR/PI3K inhibitors has been developed, starting from the consideration that the CAT sites of phosphatidylinositol-3-Kinase (PI3K) and m-TOR share a high degree of sequence homology^26^. Neutropenia and myelodysplasia arise from Sbds defects on haematopoietic and osteoprogenitor cells respectively^48^, therefore correction of hyper-activation of JAK/STAT/mTOR pathways is expected to correct both deficiencies. In conclusion, despite the fact that the precise molecular mechanism/s linking SBDS protein to ERK1/2 activation remain obscure and will represent a future subject of investigation, our findings open a wider therapeutic scenario within SDS pathology, providing a new rationale to promote the evaluation of drugs targeting mTOR pathway in SDS patients with the aim to reduce the risk to progression to bone marrow failure, myelodysplasia and leukemic transformation thus avoiding or postponing the need of bone marrow transplantation. However, in this study we focused our attention on the haematological aspect of SDS, thus not considering the other features of SDS including pancreas insufficiency, cognitive impairment and bone malformation. Our results might be the basis for *in vivo* studies using animal models mimicking to some extent SDS biological features. In this respect, it should be considered that deficiency of Sbds leads to embryonic lethality in full knockout mice. Moreover, targeting Sbds in the hematopoietic system via poly(I:C) treatment of Sbdsfl/- Tg:Mx1-cre mice resulted in a severe hepatic phenotype, preventing the use of this animal model. On the othet hand, a very recent work published by Raaijmakers^49^ and colleagues reported the generation of a new SDS mouse model based on targeted downregulation of the Sbds gene in Cebpa-expressing cells which may become suitable tools for future investigation on the mTOR/STAT3 pathway.

## Material and Methods

### Ethics Statement

All human samples used in this work were analyzed only after that written informed consent was obtained from all subjects. Methods were carried out in accordance with the approved guidelines of Ethics Committee of the AziendaOspedalieraUniversitariaIntegrata di Verona (protocol CRCFC-LymphoSDS038). All experimental protocols were approved by the Ethics Committee of the Azienda Ospedaliera Universitaria Integrata di Verona (approval nr. 658CESC).

### Patient recruitment

Nine volunteer SDS patients and nine healthy donors were recruited during the programmed Day Hospital visits for clinical evaluation at Cystic Fibrosis Centre of Verona. SDS patients have been included only if they carried the most common known SDS mutations (258 + 2T > C and 183-184TA > CT) and they did not present MDS/AML, as reported in Table 1.

Within the outpatient visit performed in the centre for routine blood chemistry, one additional blood sample (5 ml) has been obtained.

### Cell cultures

B cells were isolated from peripheral blood by Rosette Sep B Lymphocyte Kit (Miltenyi Biotech, BergischGladbach, Germany). B cell purity was verified by flow cytometry evaluating the expression of CD45 (common leucocytes antigen), CD19 (pan B lymphocytes) and by the exclusion of the expression of CD4, and CD8 molecules. B cells were seeded at a density of 3 × 10^6^ cells in 12-well cell culture plates in 3ml RPMI-1640 (Sigma-Aldrich, St Louis, MO), supplemented with 10% FBS and infected for 18 hours with EBV derived from marmoset blood leukocytes B95.8 virus-producer cell lines as previously described^50^.

### Western Blot

SBDS protein analysis: cell proteins were extracted and separated on 11% SDS-PAGE, and electroblotted onto Immobilon P filters (Millipore, Billerica, MA) previously blocked with 5% BSA in TBS (10 mMTris-HCl pH 7.4, 150 mMNaCl) supplemented with 0.05% Tween-20 (TBS/T). The membranes were probed with: i) anti-human SBDS rabbit polyclonal IgG antibody (amino acids 1 and 250 of SBDS, Abcam, Cambridge, MA, dilution 1:1500); ii) monoclonal anti-β-Actin clone AC-15 (Sigma-Aldrich, diluted 1:2000) in 1% BSA TBS/T. Membranes were incubated overnight at 4 °C and after washes, membranes were incubated with the secondary antibody, horseradish peroxidase-coupled anti-rabbit IgG (Sigma-Aldrich, dilution 1:15000), for 1 hour. Immunocomplexes were detected with ECL Plus Western Blotting detection system (Amersham Biosciences, Little Chalfont, UK). mTOR protein analysis: cell proteins were extracted and protein concentration was determined using Pierce™ BCA Protein Assay Kit (Thermo Fisher Scientific, Waltham, MA). 60 μg of cytoplasmic extracts were denatured for 5 min at 98 °C in 1x SDS sample buffer (62.5 mMTris-HCl pH 6.8, 2% SDS, 50 mM Dithiotreithol (DTT), 0.01% bromophenol blue, 10% glicerol) and loaded on SDS-PAGE gel (6% polyacrylamide) in Tris-glycine Buffer (25 mMTris, 192 mM glycine, 0.1% SDS). A *Strep*-tag protein ladder (size range of 10–250 kDa) (Precision Plus Protein WesternC Standard, Bio-Rad, Milano, Italy) was used as standard to determine molecular weight. The electrotransfer to 0.2 microns nitrocellulose membrane (Pierce, Euroclone, Milan, Italy) was performed over-night at 360 mA and 4 °C in electrotransfer buffer (10 mM CAPS pH 11, 10% methanol). The membranes were pre-stained with Ponceau S Solution (Sigma-Aldrich).Ponceau S staining was used to verify the transfer and as loading control. After that, membranes were washed with TBS for 10 min at room temperature, further washed with TBS/T and incubated in 25 ml of blocking buffer (5% Milk and TBS/T) for 1 h at room temperature. The membranes were incubated with the primary anti-mTOR (pS2448, Invitrogen, Thermo Fisher Scientific) rabbit polyclonal antibody (1:1000) in 10 ml primary antibody dilution buffer (TBS/T with 5% BSA) over-night at 4 °C. The membranes were incubated in 15 ml of blocking buffer for 1 h at room temperature, with HRP-conjugated secondary antibody (1:2000) and a StrepTactin-HRP-conjugated antibody (1:10000) used to detect protein marker. Finally, the membranes were incubated with 10 ml LumiGLO^®^ (Cell Signaling, Danvers, MA) for 5 min at room temperature and exposed to x-ray film (Pierce, Euroclone). In order to re-probe the membranes, they were stripped using the Restore™ Western Blot Stripping Buffer (Pierce, Euroclone) and incubated with other primary (anti-mTOR, Cell Signalling) and secondary antibodies. The chemiluminescent signal was visualized on X-ray films and the intensity of the immunopositive bands was analyzed by Gel Doc 2000 (Bio-Rad)using Quantity One program to elaborate the intensity data of our specific target protein.

### Human Phospho Kinase Array

LCLs were seeded at a density of 2.5 × 10^6^ cells in 6-wells plates containing RPMI-1640 supplemented with 0.5% FBS and incubated for 24 hours at 37 °C, in order to synchronize cell growth. Subsequently, 9.5% FBS was added to each well and cells were incubated in the presence or in the absence of 10 ng/ml of human recombinant IL-6 (R&D systems, Minneapolis, MN) for 15 min. Cells were then lysed and total proteins were extracted in the presence of Complete EDTA-free Protease Inhibitor Cocktail Tablets (Roche, Penzberg, Germany). According to the manufacturer’s protocol, 200 μg cell lysate was incubated with each human phospho-MAPK array (R&D Systems, Minneapolis, MN). Cell lysates were diluted and incubated overnight with nitrocellulose membranes in which capture and control antibodies against 43 different kinases and transcription factors, have been spotted in duplicate. The arrays were washed to remove unbound proteins and were incubated with a cocktail of biotinylated detection antibodies. Streptavidin-HRP and chemiluminescent detection reagents were applied and a signal was produced at each capture spot corresponding to the amount of phosphorylated protein bound. Pixel densities on developed X-ray film were collected and analyzed using a transmission mode scanner and Digimizer image analysis software (MedCalc software, Ostend, Belgium).

### Flow cytometry

LCLs were seeded at 2.5 × 10^5^ cells in 4 aliquots and incubated at 37 °C in the presence or in the absence of 350 nM Rapamycin (Sigma-Aldrich, St Louis, MO) for 1 hour. Then cells were stimulated with IL-6 (10 ng/ml) for further 15 minutes. LCLs were fixed in 2% paraformaldehyde and permeabilized in 100% ice-cold methanol, washed twice in flow buffer (PBS, pH 7.2, with 0.2% BSA and 0.09% sodium azide) as previously described^51^ and then stained with anti-pS727-STAT3-PE, anti-Y705-STAT3-PE, anti-p-S2448-mTOR-PE or isotype control-PE for 30 minutes (antibodies were purchased by Becton-Dickinson Biosciences, Franklin Lakes, NJ). Peripheral blood sample derived from SDS patients or healthy subjects (3 ml each) were used within 2 hours after blood withdrawal. Plasma was separated from whole blood by centrifugation at 1500 × *g* for 10 min. Red blood cells were lysed in 40 ml of solution containing 0.89% (w/v) NH4Cl, 0.10% (w/v) KHCO_3_ and 200 μM EDTA as previously reported^52^. Leukocytes were cultured in 6-wells plate containing RPMI-1640 supplemented with 10% freshly prepared, heat-inactivated human plasma. Cells were stained with APC 700 CD45, APC750 CD3 and KRO CD19 conjugated antibodies and incubated in the presence or in the absence of IL-6 (10 ng/ml) for 15 min, centrifuged at low speed (600 × *g*) for 10 min, washed with ice-cold PBS, then fixed and permeabilized with Intracellular Fixation and Permeabilization Buffer Set (eBioscience, San Diego, CA), following the manufacturer’s protocol. After permeabilization, both LCLs and primary leukocytes were washed once in flow buffer and stained with anti-pS727-STAT3-PE, anti-Y705-STAT3-PE, anti-p-S2448-mTOR-PE or isotype control-PE conjugated antibodies for 30 minutes. Cells were washed and acquired on a 10 color, 3 laser (Blue Solid State Diode: 488 nm, 22 mW, Red Solid State Diode: 638 nm, 25 mW, Violet Solid State Diode: 405 nm, 40 mW), Navios flow cytometer (Beckman Coulter, Indianapolis, IN). All acquired data files were analyzed using the “Navios” or Kaluza software, version 1.3 (Beckman Coulter, Indianapolis, IN). CD45 versus SS gating strategy was used to recovery lymphocytes, monocytes and neutrophils regions. Lymphocytes region was plotted on CD3 versus CD19 dotplot to isolate B cells population.

### STATs nuclear translocation assay

Human Phospho-STAT Family Trans-AM kit (Active Motif, Carlsbad, CA), which detects the phosphorylated form of active STAT3 has been performed. We produced nuclear extracts as previously reported^53^. Briefly, 2 × 10^6^ LCLs were incubated in RPMI-1640 medium containing 0.5% FBS for 24 hours, in order to synchronize cell growth. Cells were subsequently incubated in the presence or in the absence of IL-6 (10 ng/ml) in RPMI-1640 supplemented with 10% FBS, washed with ice-cold PBS, pelleted and resuspended in 400 μl of cold buffer containing 10 mM HEPES-KOH pH 7.9, 1.5 mM MgCl2, 10 mMKCl, 0.5 mM DTT, 0.2 mM PMSF). Samples were centrifuged at 13,000 rpm for 10 sec, and the supernatants fraction was discarded. The pellets were resuspended in 50 μl of ice-cold buffer containing 20 mM HEPES-KOH pH 7.9, 25% glycerol, 420 mMNaCl, 1.5 mM MgCl_2_, 0.2 mM EDTA, 0.5 mM DTT, 0.2 mM PMSF and incubated on ice for 20 min for high-salt extraction. Cellular debris were removed by centrifugation at 13,000 rpm for 2 min at 4 °C and the supernatant fractions, containing DNA-binding proteins, were stored at −80 °C. The Human Phospho-STAT family Trans-AM kit contains a 96-wells plate on which has been immobilized oligonucleotides containing the STAT1, STAT3, STAT5A and STAT5B consensus sequences. The active STATs contained in nuclear extracts specifically bind to these sequences. Human Phospho STAT Family Trans-AM kit was performedusing 2.5 μg nuclear extracts for each sample, according to the manufacturer’s instructions. The binding was revealed by subsequent addition of primary antibodies directed against STAT isoforms and a secondary Ab conjugated to HRP. After the addition of the substrate, absorbance was read at a 450-nm wavelength.

### SBDS gene silencing

SBDS gene silencing was performed using TriFECTa RNAi Kit (Integrated DNA technologies, Coralville, Iowa, IA), accordingly to the manufacturer’s instructions. LCLs derived from healthy donors were transiently transfected with 2 different specific siRNA sequences for SBDS, or with scrambled sequence as control. siRNA oligonucleotides were complexed with cationic liposomes siPORT Neo-FX (Thermo Fisher Scientific, Waltham, MA). Briefly, 0.75 μl of siPORT Neo-FX were complexed with siRNA or Scrambled oligos (40 mM each) in 1 ml RPMI-1640 medium supplemented with 0.5% FBS and added to each well of a 12-well plate containing 2.5 × 10^5^ cells. In order to check the efficiency rate of transfection, a PE-conjugated siRNA was produced, complexed with liposomal vector as previously described and incubated in LCLs. Plates were incubated for 24 hours at 37 °C. Knock-down of SBDS gene expression was determined by western blot analysis.

### Phospho-mTOR S2448 ELISA

Analysis of phospho-mTOR was performed by using the PathScanPhospho-mTOR (Ser2448) Sandwich ELISA Kit (Cell Signaling, Danvers, MA), following the manufacturer’s protocol. Briefly, 2.5 × 10^6^ LCLs were seeded in 12-wells plates containing RPMI-1640 medium supplemented with 0.5% FBS and incubated at 37 °C for 24 hours in order to reach cell growth synchronization. Then, cells were incubated in the presence or in the absence of i) siRNA/Scrambled:siPort Neo FX complexes for 24 hours; ii) ERK inhibitor U0126 (10 μM), or vehicle alone (DMSO) for 1 hour as previously reported^31^. Subsequently, cells were washed, lysed with Lysis Buffer containing 1 mM PMSF (Sigma-Aldrich, St Louis, MO) and Protease Inhibitor Cocktail Tablets (Roche, Penzberg, Germany). Protein extracts (25 mg/ml) were added to each well of ELISA plate, and incubated overnight at 4 °C. 100 μl of rabbit detection antibody against mTOR S2448 were added to each well and incubated at 37 °C for 1 hour. Anti-rabbit IgG, HRP-linked Antibody was then used to recognize the bound detection antibody. HRP substrate TMB was added to develop color. The magnitude of the absorbance for this developed color was proportional to the amount of mTOR phosphorylated at Ser2448.

### Cell proliferation assay

Cellular proliferation was tested by XTT Cell Proliferation Kit II (Roche, Penzberg, Germany). The assay is based on the cleavage of yellow tetrazolium salt XTT to form an orange formazan dye by metabolic active cells. The formation of the dye is quantified using a scanning multiwell spectrophotometer (ELISA reader). LCLs were synchronized by incubation for 24 hours in RPMI-1640 medium containing 0.5% FBS. After this period, cells were seeded at a density of 5 × 10^4^ cells/well in 96 multiwells plates in 100 μl/well RPMI-1640 medium containing 10% FBS in the presence or in the absence of 350 nM mTOR inhibitor Rapamycin (Sigma-Aldrich) or 10 μM STAT-3 inhibitor STATTIC (Sigma-Aldrich). Cell proliferation was stimulated with increasing doses of IL-6 (0.01–10 ng/ml) for 48 hours. Finally, 100 μl of yellow tetrazolium salt was added to each well and plates were incubated for 4 hours at 37 °C. The increase in number of living cells directly correlated to the amount of orange formazan formed, which has been monitored by absorbance at 450 nm.

### Apoptosis Analysis

LCLs cells (2.5 × 10^5^) were seeded in 24 wells plate in RPMI-1640 medium supplemented with 0.5% FBS and incubated at 37 °C and 5% CO_2_ over night in order to reach the cell growth synchronization. The day after, FBS was added to the final concentration of 10% and cells were incubated for 24 hours in presence or absence of 350 nM Rapamycin or 20 μM STATTIC inhibitors.

Apoptosis was analyzed by Annexin V that detect the phophatidyl-serine (PS) exposed on the external membrane of the apoptotic cells. Annexin V analysis was performed using the Muse Annexin V & Dead Cell Kit (Millipore) according to the manufacturer instruction. 100 μl of cells were mixed with the same volume of ready-to-use Muse Annexin V & Dead Cell Reagent, and incubated for 20 minutes at room temperature in the dark. Sample were loaded into the Muse instrument (Millipore) and data were analyzed with Muse Annexin V & Dead Cell Software Module.

### Statatistical analysis

Descriptive statistics were reported for the following variables: SDS UT, CTL UT, CTL IL6, SDS IL6, SDS U0126, SDS IL6, SDS IL6 U0126, sir1, sir2, IL6, sir1IL6, and sir2IL6.

Independent group were tested using Mann-Whitney test, while Wilcoxon signed-rank test or Student’s t-test were used in case of paired data. A p-value < 0.05 was considered statistically significant. The statistical software SAS (SAS Institute Inc., Cary, NC) and SigmaPlot (Systat Software Inc., San Jose, CA) were used.

## Additional Information

**How to cite this article**: Bezzerri, V. *et al*. New insights into the Shwachman-Diamond Syndrome-related haematological disorder: hyper-activation of mTOR and STAT3 in leukocytes. *Sci. Rep.* **6**, 33165; doi: 10.1038/srep33165 (2016).

## Supplementary Material

Supplementary Information

## Figures and Tables

**Table 1:** Clinical details of SDS patients enrolled in this study.

Subject	Age	Gender	Genotype	MDS	AML	PMN/mm3
SDS1	13	M	258 + 2T > C/183-184TA > CT	No	No	280
SDS2	37	M	258 + 2T > C/183-184TA > CT	No	No	1700
SDS3	9	M	258 + 2T > C/183-184TA > CT	No	No	430
SDS4	38	M	258 + 2T > C/183-184TA > CT	No	No	390
SDS5	23	F	258 + 2T > C/183-184TA > CT	No	No	1340
SDS6	7	M	258 + 2T > C/183-184TA > CT	No	No	910
SDS7	9	M	258 + 2T > C/183-184TA > CT	No	No	890
SDS8	23	F	258 + 2T > C/183-184TA > CT	No	No	300
SDS9	10	F	258 + 2T > C/183-184TA > CT	No	No	200
