# Supplementary material for: New insights into the Shwachman-Diamond Syndrome-related haematological disorder: hyper-activation of mTOR and STAT3 in leukocytes
Source: Sci Rep. 2016 Sep 23;6:33165. doi: 10.1038/srep33165 (PMC5034238; doi:10.1038/srep33165)
Supplement: Supplementary Information [file srep33165-s1.pdf]

New insights into the Shwachman-Diamond Syndrome-related haematological disorder: hyper-activation of mTOR and STAT3 in leukocytes.

Valentino Bezzerri, Antonio Vella, Elisa Calcaterra, Alessia Finotti, Jessica Gasparello, Roberto Gambari, Baroukh Maurice Assael, Marco Cipolli and Claudio Sorio

SUPPLEMENTARY INFORMATION

Supplementary Figure legends

Supplementary figure S1

SBDS protein expression in LCLs.

Western blot analysis to evaluate SBDS protein expression in LCLs. LY52 and LY53: LCLs derived from healthy control subjects. LY190, LY193, LY198: LCLs obtained from SDS patients carrying genotype 258+2T>C / 183-184TA>CT.

Supplementary figure S2

Western Blot analysis of mTOR S727 phosphorylation in LCLs.

Increase of S2448 phosphorylated mTOR in SDS. **a)** western blotting analysis using the polyclonal rabbit antibody recognizing the S2448 phosphorylated form of mTOR (arrowed). **b)** representative western blotting analysis of protein extracts from a control subject (on the left) and a SDS patient (on the right) and summary of the relative content of the S2448 phosphorylated mTOR in control (open box) and SDS-derived LCLs (black box). Data are mean ± SD of two independent experiments performed in duplicate. Student's t test has been reported. **c)** representative western blotting analysis of protein extracts from SDS LCLs culture in the absence or in the presence of IL-6, with (+) or without (-) 350 nM rapamycin. **d)** summary of the relative content of the S2448

phosphorylated mTOR in control (open box) and SDS patients (black box). Data represent the % values relative to untreated cells. The S2448 phosphorylated mTOR is presented in control (open box) and SDS-derived LCLs induced (+) or not (-) with IL-6. Data are mean ± SD of two independent experiments performed in duplicate. Student's t test has been reported.

Supplementary figure S3

Effect of SBDS gene silencing in LCLs on mTOR S2448 phosphorylation and role of ERK1/2 MAPK. **a)** LCLs derived from healthy donors were transiently transfected with 2 different specific siRNA sequences (siRNA 1 and siRNA 2) for SBDS, or with PE-conjugated siRNA sequence, or with scrambled sequence as control in the presence of cationic liposomal vector for 24 hours. mTOR S2448 phosphorylation was detected using PathScan phospho-mTOR ELISA kit. Data are mean ± SEM of 5 independent experiments performed in 3 different healthy control cell lines, in duplicate. **b)** Effect of pre-incubation of ERK inhibitor U0126 (10 μM) in SDS-derived LCLs for 1 hour before stimulation with IL-6 (10 ng/ml) for further 15 min. Data are mean ± SEM of 6 independent experiments performed in 3 different healthy control cell lines, in duplicate. Wilcoxon signed-rank test has been calculated.

Supplementary figure S4

Flow cytometric analysis of mTOR S2448 phosphorylation in primary leukocytes. Representative experiment conducted in leukocytes derived from peripheral blood of a SDS patient carrying genotype 258+2T>C/183-184TA>CT compared to a healthy control subject. **a)** morphological distribution of CD45 expressing leukocytes in healthy donor derived peripheral blood. **b)** morphological distribution of CD45 expressing leukocytes in SDS patient derived peripheral blood. PMNs, Monocytes and Lymphocytes are gated. **c,d)** B cell (CD19+) region isolated from lymphocytes regions of panels A and B, respectively, plotted on CD3 versus CD19 dotplot . **e)** mTOR S2448 phosphorylation observed in B cells in the presence or in the absence (UT) of IL-6 stimulation (10 ng/ml) for 15 min. **f)** mTOR S2448 phosphorylation observed in PMNs in the presence or in the absence of IL-6 stimulation (10 ng/ml) for 15 min. **g)** mTOR S2448

phosphorylation observed in Monocytes in the presence or in the absence of IL-6 stimulation (10 ng/ml) for 15 min.

Supplementary figure S5

Effect of rapamycin on mTOR S2448 phosphorylation in primary leukocytes.

Primary leukocytes derived from two patients carrying genotype 258+2T>C / 183-184TA>CT and from two healthy control subjects were incubated in the presence or in the absence of rapamycin for 1 hour before IL-6 stimulation (10 ng/ml) for further 15 min and analyzed by flow cytometry. **a,b)** MFI of mTOR S2448 phosphorylation measured in primary B cells derived from healthy control and SDS patient, respectively. **c,d)** MFI of mTOR S2448 phosphorylation measured in primary PMNs derived from healthy control and SDS patient, respectively. **e,f)** MFI of mTOR S2448 phosphorylation measured in primary monocytes derived from healthy control and SDS patient, respectively. Data are mean ± SEM of two independent experiments performed in LCLs obtained from three different SDS patients and compared to three different healthy donors. Student's t test has been calculated.

Supplementary figure S6

Flow cytometric analysis of STAT3 Y705 and S727 phosphorylation in primary leukocytes.

Representative experiment conducted in leukocytes derived from peripheral blood cells of a SDS patients carrying genotypes 258+2T>C / 183-184TA>CT compared to a healthy control subject. **a)** STAT3 phosphorylation observed in B cells in the presence or in the absence (UT) of IL-6 stimulation (10 ng/ml) for 15 min. **b)** STAT3 phosphorylation observed in PMNs in the presence or in the absence of IL-6 stimulation (10 ng/ml) for 15 min. **c)** STAT3 phosphorylation observed in Monocytes in the presence or in the absence of IL-6 stimulation (10 ng/ml) for 15 min.

Supplementary figure S7

Effect of rapamycin on STAT3 Y705 phosphorylation in primary leukocytes.

Primary leukocytes derived from two patients carrying genotype 258+2T>C / 183-184TA>CT and from two healthy control subjects were incubated in the presence or in the absence of rapamycin for 1 hour before IL-6 stimulation (10 ng/ml) for further 15 min and analyzed by flow cytometry. **a,b)** MFI of STAT3 Y705 phosphorylation measured in primary B cells derived from healthy control and SDS patient, respectively. **c,d)** MFI of STAT3 Y705 phosphorylation measured in primary PMNs derived from healthy control and SDS patient, respectively. **e,f)** MFI of STAT3 Y705 phosphorylation measured in primary monocytes derived from healthy control and SDS patient, respectively. Data are mean ± SEM of two independent experiments performed in LCLs obtained from three different SDS patients and compared to three different healthy donors. Student's t test has been calculated.

Supplementary figure S8

Effect of rapamycin on STAT3 S727 phosphorylation in primary leukocytes.

Primary leukocytes derived from two patients carrying genotype 258+2T>C / 183-184TA>CT and from two healthy control subjects were incubated in the presence or in the absence of rapamycin for 1 hour before IL-6 stimulation (10 ng/ml) for further 15 min and analyzed by flow cytometry. **a,b)** MFI of STAT3 S727 phosphorylation measured in primary B cells derived from healthy control and SDS patient, respectively. **c,d)** MFI of STAT3 S727 phosphorylation measured in primary PMNs derived from healthy control and SDS patient, respectively. **e,f)** MFI of STAT3 S727 phosphorylation measured in primary monocytes derived from healthy control and SDS patient, respectively. Data are mean ± SEM of two independent experiments performed in LCLs obtained from three different SDS patients and compared to three different healthy donors. Student's t test has been calculated.

SUPPLEMENTARY FIGURES

Figure S1

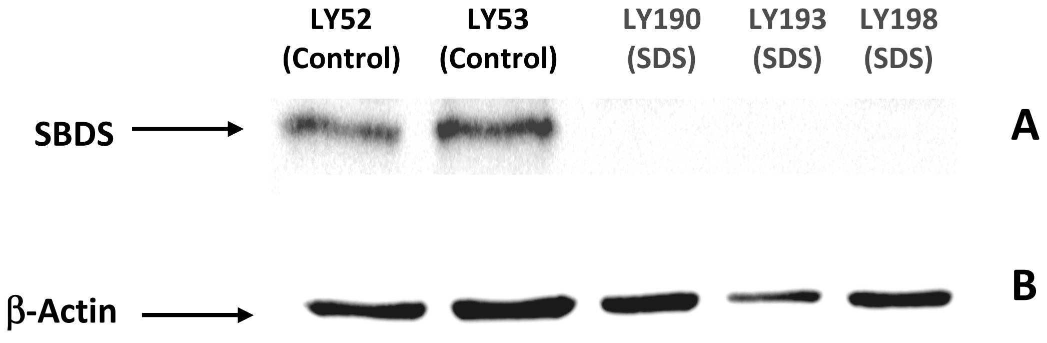

Figure S2

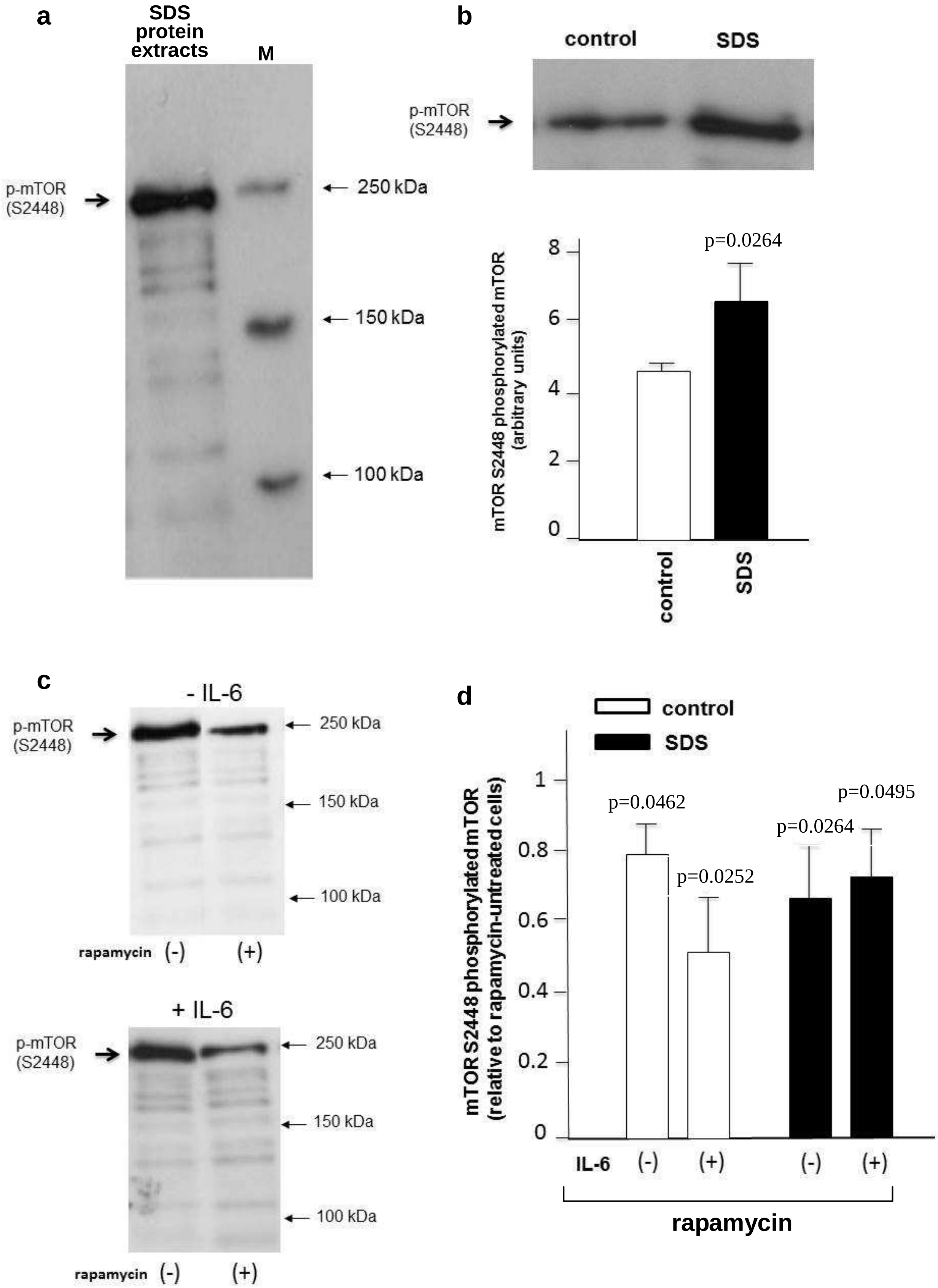
a
SDS protein extracts
M
p-mTOR (S2448)
250 kDa
150 kDa
100 kDa
b
control
SDS
p-mTOR (S2448)
p=0.0264
mTOR S2448 phosphorylated mTOR (arbitrary units)
0
2
4
6
8
control
SDS
c
- IL-6
p-mTOR (S2448)
250 kDa
150 kDa
100 kDa
rapamycin (-) (+)
+ IL-6
p-mTOR (S2448)
250 kDa
150 kDa
100 kDa
rapamycin (-) (+)
d
control
SDS
mTOR S2448 phosphorylated mTOR (relative to rapamycin-untreated cells)
0
0.2
0.4
0.6
0.8
1
p=0.0462
p=0.0252
p=0.0264
p=0.0495
IL-6 (-) (+) (-) (+)
rapamycin

Figure S3

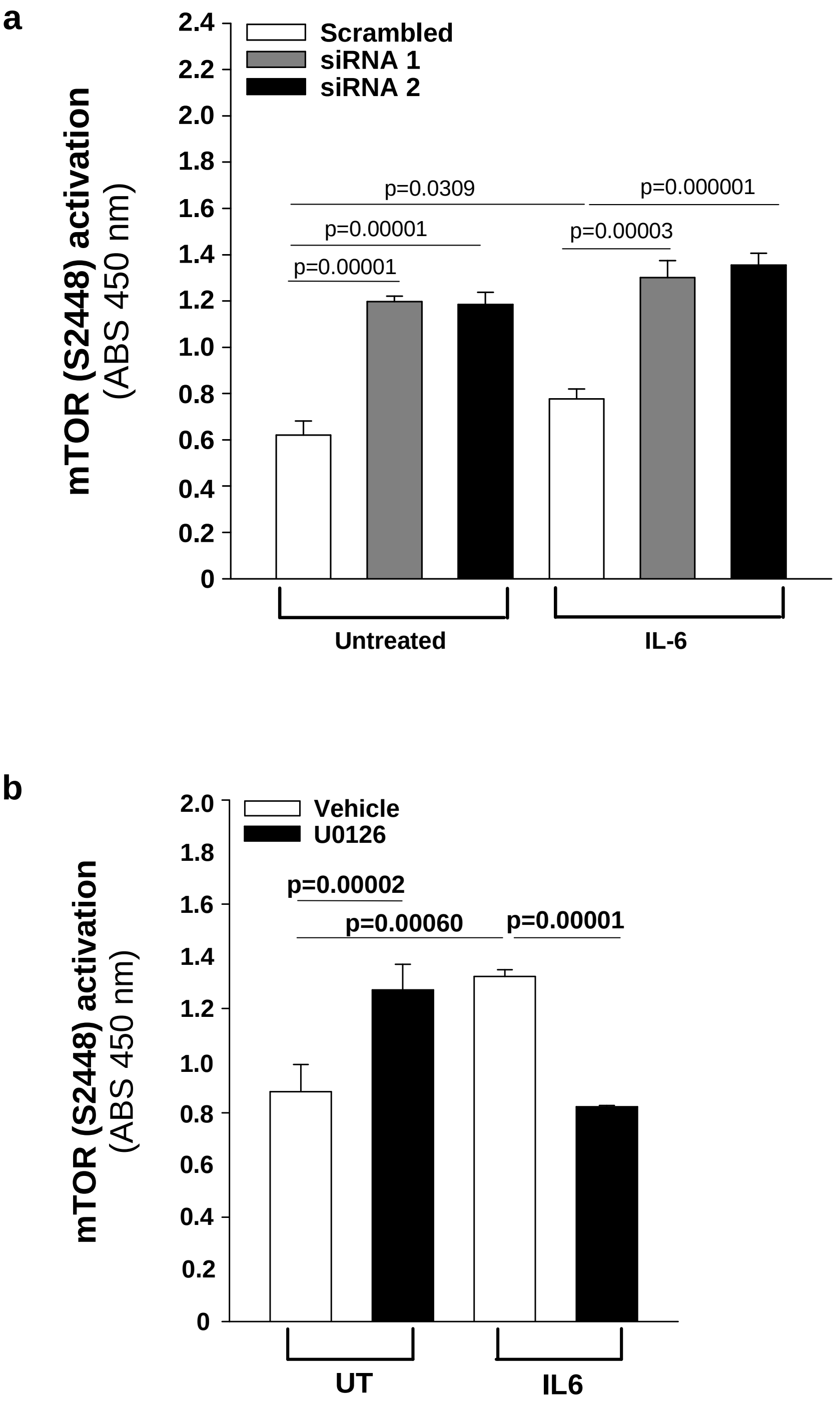
a
mTOR (S2448) activation
(ABS 450 nm)
2.4
2.2
2.0
1.8
1.6
1.4
1.2
1.0
0.8
0.6
0.4
0.2
0
Scrambled
siRNA 1
siRNA 2
p=0.0309
p=0.000001
p=0.00001
p=0.00003
p=0.00001
Untreated
IL-6
b
mTOR (S2448) activation
(ABS 450 nm)
2.0
1.8
1.6
1.4
1.2
1.0
0.8
0.6
0.4
0.2
0
Vehicle
U0126
p=0.00002
p=0.00060
p=0.00001
UT
IL6

Figure S4

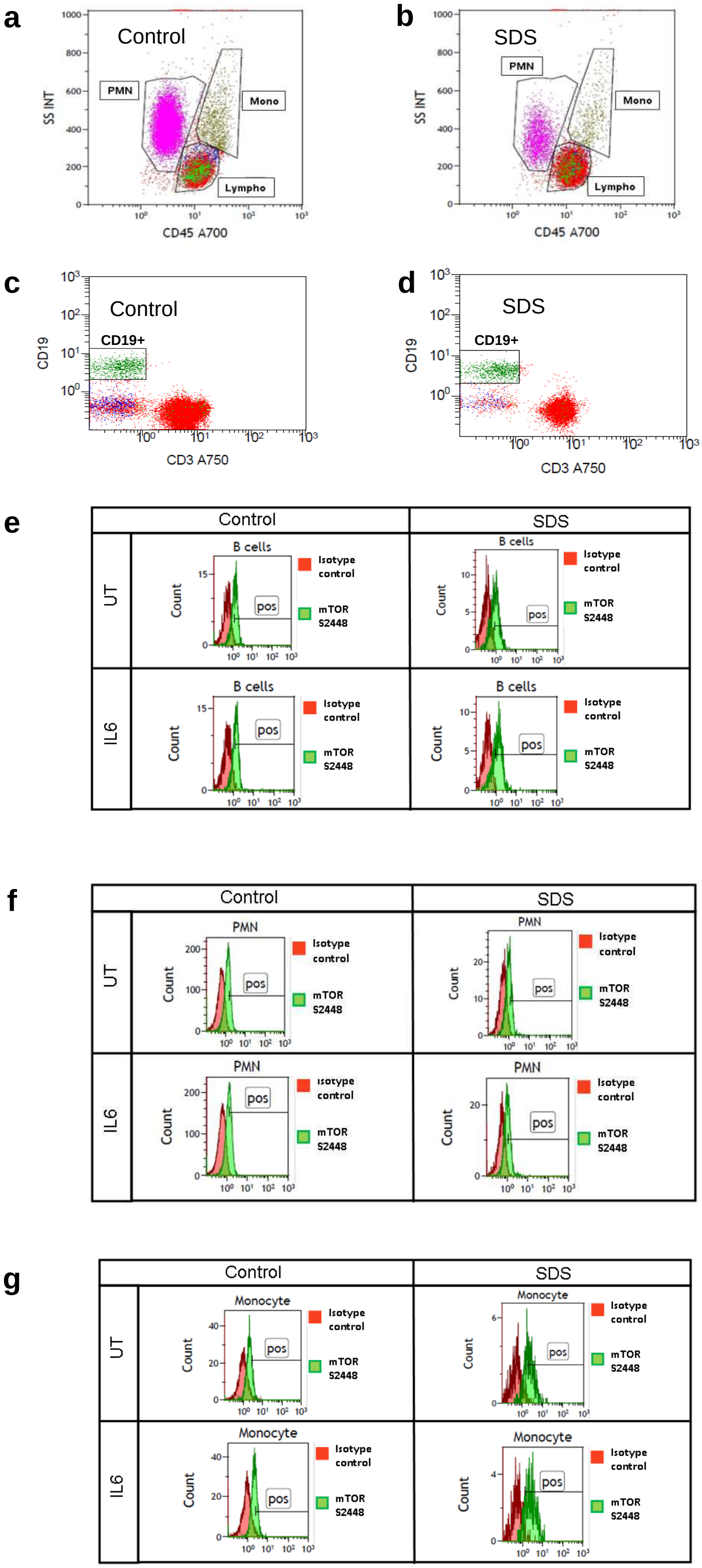
a
Control
PMN
Mono
Lympho
SS INT
CD45 A700
b
SDS
c
CD19+
CD19
CD3 A750
d
e
UT
IL6
B cells
Count
pos
Isotype control
mTOR S2448
f
PMN
g
Monocyte

Figure S5

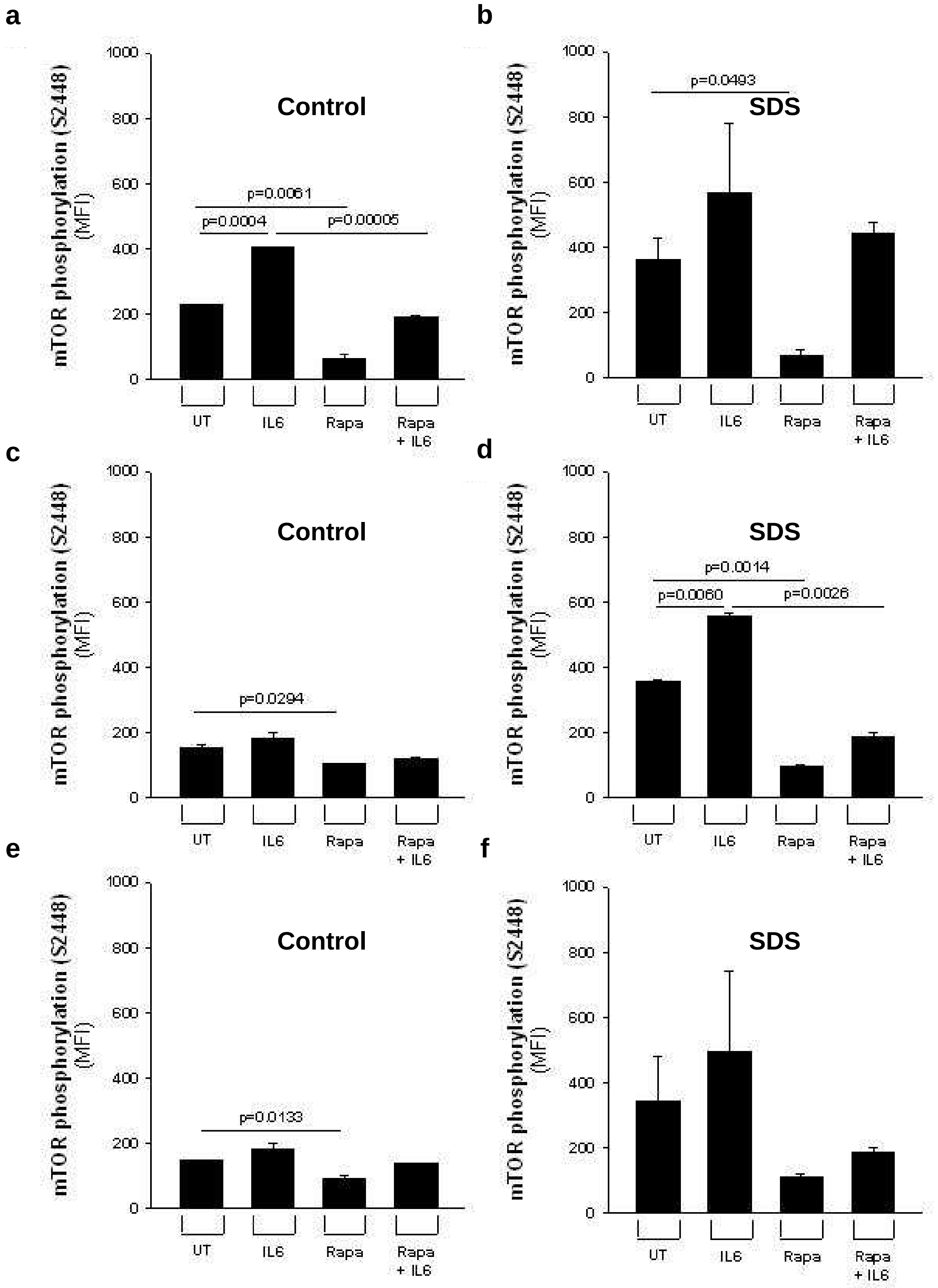
a
b
c
d
e
f
Control
SDS
mTOR phosphorylation (S2448) (MFI)
1000
800
600
400
200
0
p=0.0061
p=0.0004
p=0.00005
p=0.0493
p=0.0294
p=0.0014
p=0.0060
p=0.0026
p=0.0133
UT
IL6
Rapa
Rapa + IL6

Figure S6

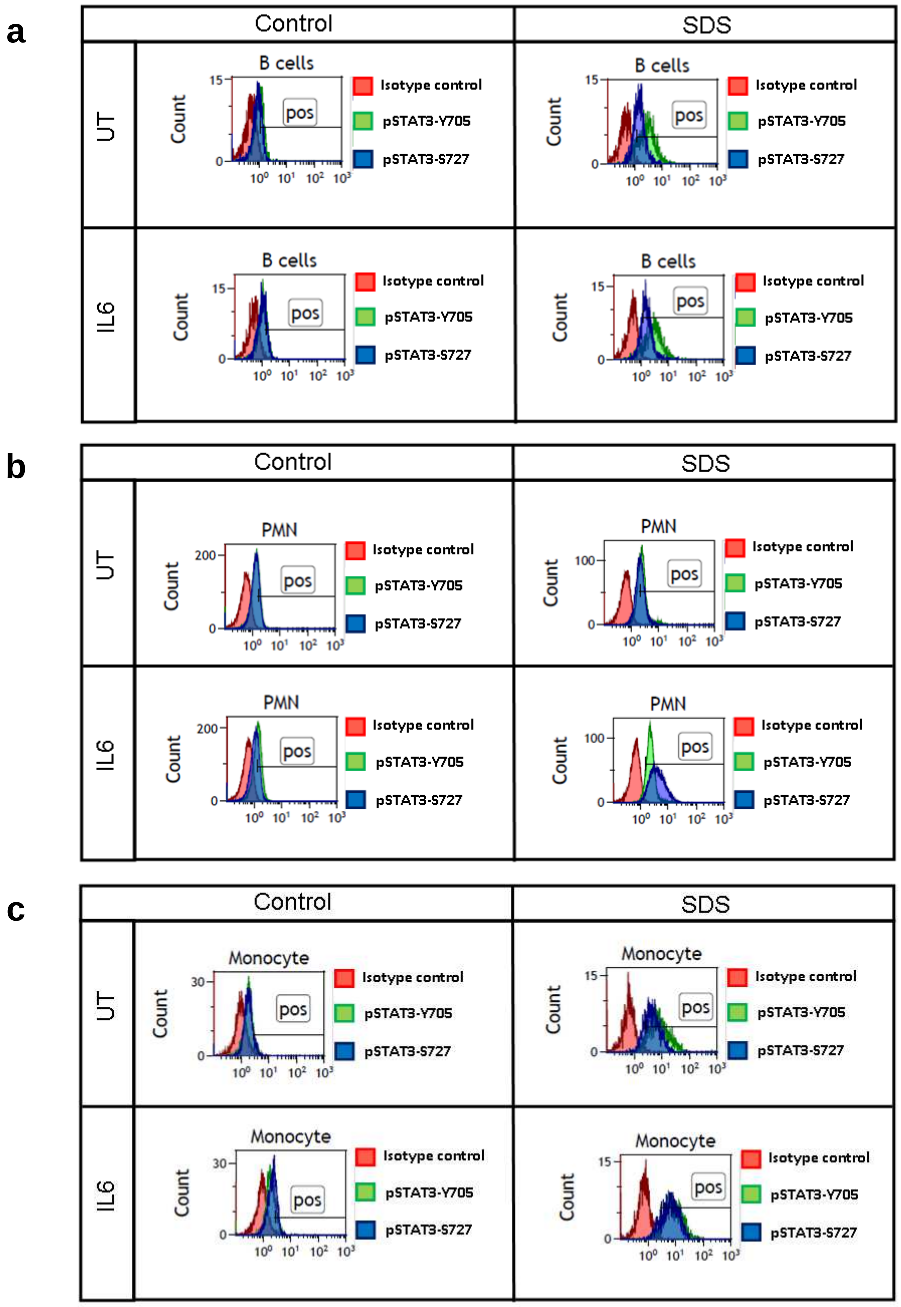
a
Control
SDS
UT
IL6
B cells
Count
pos
Isotype control
pSTAT3-Y705
pSTAT3-S727
b
PMN
c
Monocyte

Figure S7

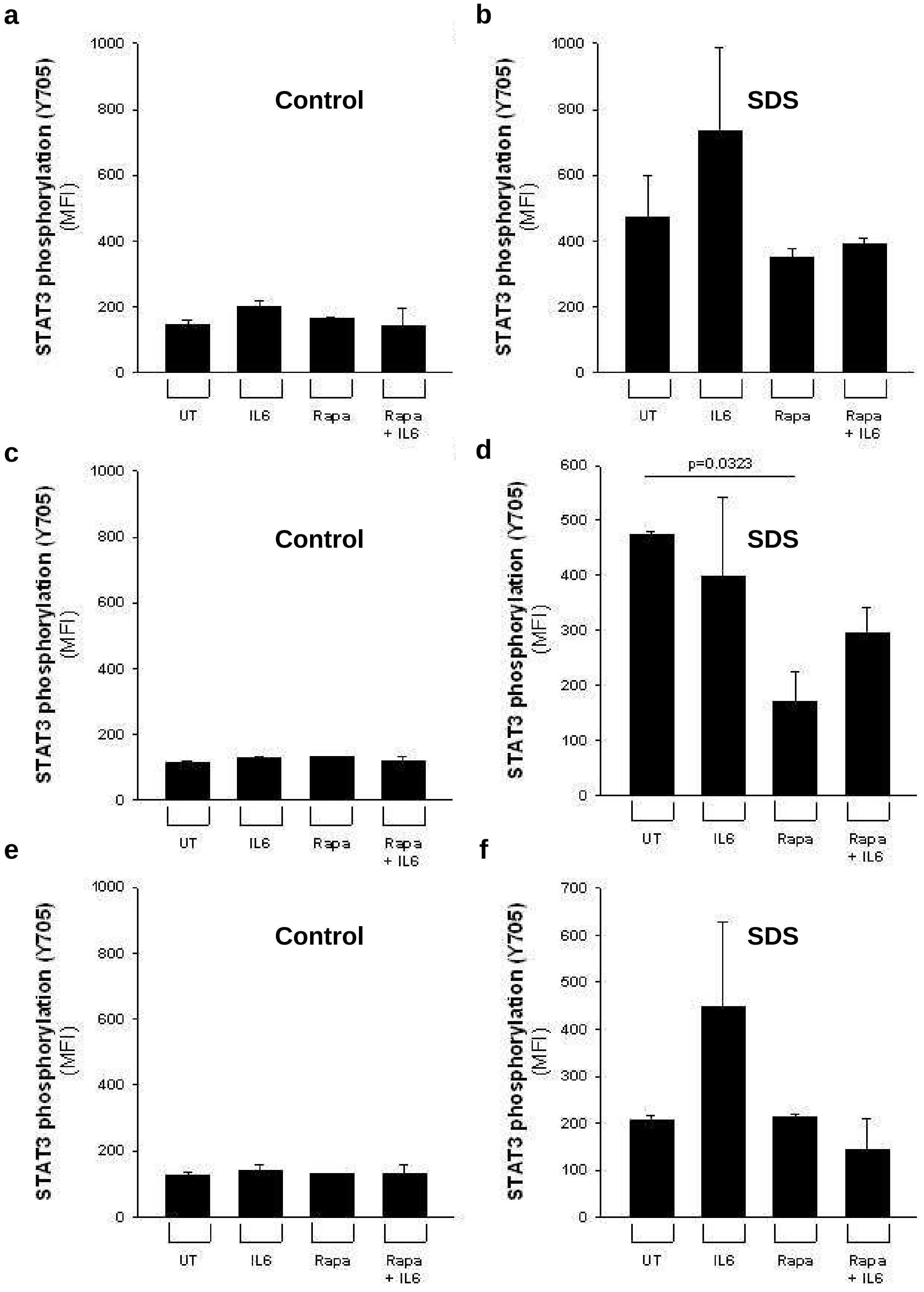
a
STAT3 phosphorylation (Y705) (MFI)
Control
0
200
400
600
800
1000
UT
IL6
Rapa
Rapa + IL6
b
STAT3 phosphorylation (Y705) (MFI)
SDS
0
200
400
600
800
1000
UT
IL6
Rapa
Rapa + IL6
c
STAT3 phosphorylation (Y705) (MFI)
Control
0
200
400
600
800
1000
UT
IL6
Rapa
Rapa + IL6
d
STAT3 phosphorylation (Y705) (MFI)
p=0.0323
SDS
0
100
200
300
400
500
600
UT
IL6
Rapa
Rapa + IL6
e
STAT3 phosphorylation (Y705) (MFI)
Control
0
200
400
600
800
1000
UT
IL6
Rapa
Rapa + IL6
f
STAT3 phosphorylation (Y705) (MFI)
SDS
0
100
200
300
400
500
600
700
UT
IL6
Rapa
Rapa + IL6

Figure S8

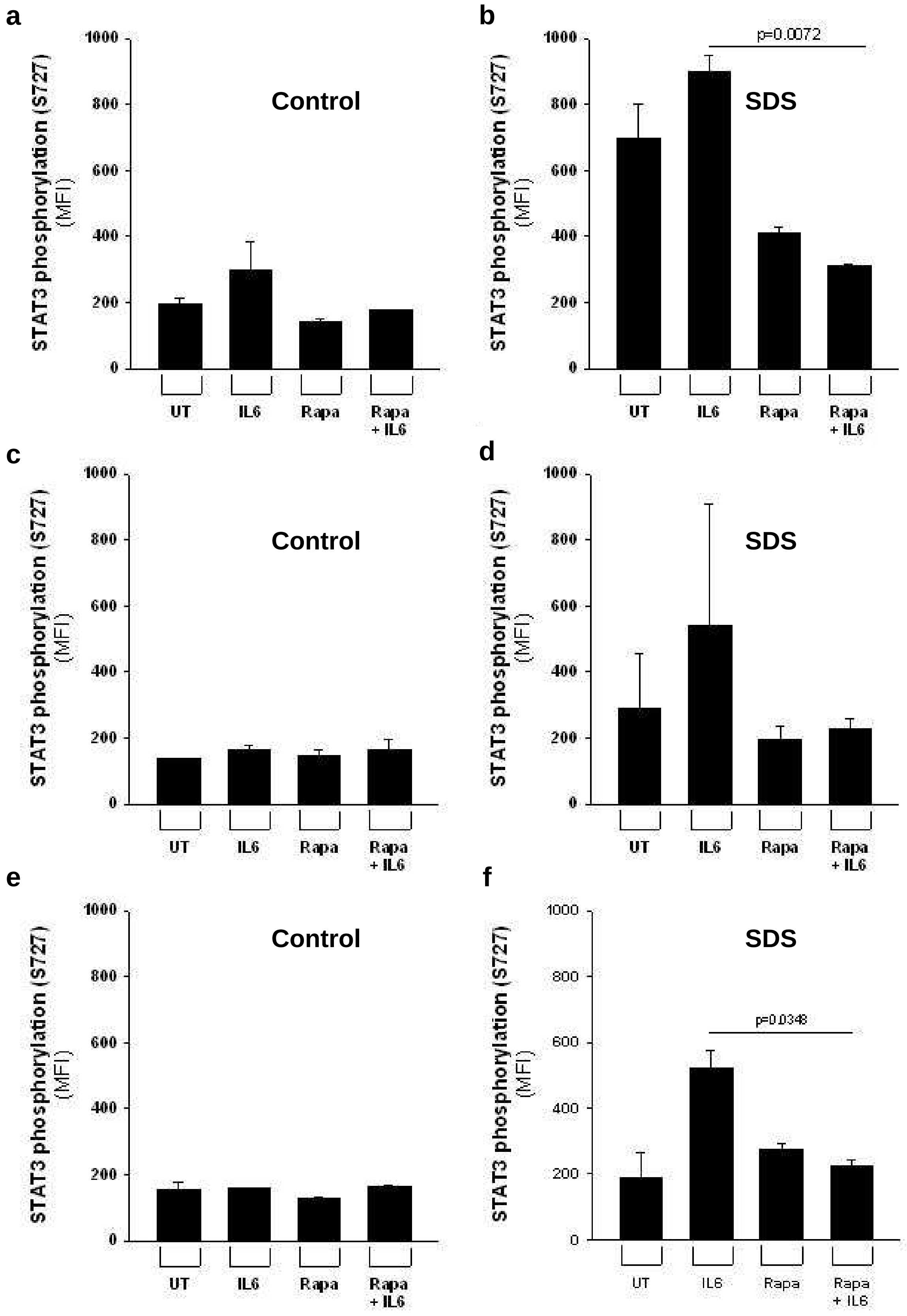
a
STAT3 phosphorylation (S727) (MFI)
1000
800
600
400
200
0
Control
UT
IL6
Rapa
Rapa + IL6
b
STAT3 phosphorylation (S727) (MFI)
p=0.0072
SDS
UT
IL6
Rapa
Rapa + IL6
c
Control
d
SDS
e
Control
f
SDS
p=0.0348
